# Two-Parameter Modified Ridge-Type M-Estimator for Linear Regression Model

**DOI:** 10.1155/2020/3192852

**Published:** 2020-05-15

**Authors:** Adewale F. Lukman, Kayode Ayinde, B. M. Golam Kibria, Segun L. Jegede

**Affiliations:** ^1^Department of Physical Sciences, Landmark University, Omu-Aran, Nigeria; ^2^Department of Statistics, Federal University of Technology, Akure, Nigeria; ^3^Department of Mathematics and Statistics, Florida International University, Miami, FL, USA

## Abstract

The general linear regression model has been one of the most frequently used models over the years, with the ordinary least squares estimator (OLS) used to estimate its parameter. The problems of the OLS estimator for linear regression analysis include that of multicollinearity and outliers, which lead to unfavourable results. This study proposed a two-parameter ridge-type modified M-estimator (RTMME) based on the M-estimator to deal with the combined problem resulting from multicollinearity and outliers. Through theoretical proofs, Monte Carlo simulation, and a numerical example, the proposed estimator outperforms the modified ridge-type estimator and some other considered existing estimators.

## 1. Introduction

A multiple linear regression model can be defined mathematically as

(1) $$\begin{array}{c} y=X\beta +\varepsilon , \end{array}$$

where *y* is an *n* × 1 vector of observations referred to as the dependent variable; *X* is a known full column rank of *n* × *p*standardized and centered explanatory variable matrix; *β*  is an *p* × 1 vector of unknown parameters; and *ε* is an *n* × 1 vector of disturbances with *E*(*ε*) = 0 and dispersion matrix Cov(*ε*) =  *σ*^2^*I*, *I* is the *n* × *n* identity matrix. The ordinary least squares estimator (OLSE) of *β* is given as

(2) $$\begin{array}{c} \hat{\beta }={\left(X^{\prime }X\right)}^{-1}X^{\prime }y. \end{array}$$

According to Gauss–Markov theorem, OLS estimator is the best linear unbiased estimator (BLUE) possessing minimum variance in the class of all unbiased linear estimators [1, 2]. However, the performance of the estimator is imprecise in the presence of multicollinearity [3]. Biased estimators such as ridge regression estimator [4], Liu estimator [5] and Stein estimator [6], principal component estimator [7], modified ridge regression estimator [8], and others are often employed to tackle this problem. Another factor whose presence can negatively influence the regression coefficients of the OLS estimator is the outlier. The general practice in the literature is that one adopts robust estimators as an alternative to the OLS estimator. The M-estimator is popularly used to handle outlier in the *y*-direction [9].

Hoerl and Kennard [4] defined the ridge estimator (RE) as

(3) $$\begin{array}{c} \hat{\beta }\left(k\right)={\left(X^{\prime }X+kI\right)}^{-1}X^{\prime }y=R_{k}\hat{\beta }, \end{array}$$

where *R*_*k*_=(*X*′*X*+*kI*)^−1^*X*′*X* and *k* > 0. However, RE can be sensitive to outliers in the *y*-direction; a remedial measure is the ridge M-estimator (RME) suggested by Silvapulle [10] as

(4) $$\begin{array}{c} {\hat{\beta }}_{M}\left(k\right)=R_{k}{\hat{\beta }}_{M}, \end{array}$$

where ${\hat{\beta }}_{M}$ is the M-estimator of *β* [11].

Dorugade [12] modified the ridge estimator and defined it as

(5) $$\begin{array}{c} {\hat{\beta }}_{D}\left(k\right)=R_{kd1}\hat{\beta }, \end{array}$$

where *R*_*kd*1_ = (*X*′*X* + *kdI*)^−1^*X*′*X* and *d* introduced as an additional basing parameter.

Following Dorugade [12], Lukman et al. [13] modified the ridge estimator in (3) and called it the modified ridge-type estimator (MRT). The estimator is defined as

(6) $$\begin{array}{c} {\hat{\beta }}_{\text{MRT}}\left(k,d\right)={\left(X^{\prime }X+k\left(1+d\right)I\right)}^{-1}X^{\prime }Y=R_{kd}\hat{\beta }, \end{array}$$

where *R*_*kd*_=(*X*′*X*+*k*(1+*d*)*I*)^−1^*X*′*X*.

The organization of the paper is as follows. We proposed the new estimator in Section 2 and provided a theoretical comparison among the estimators in Section 3. We discussed the robust choice of the biasing parameters in Section 4 and conducted simulation studies in Section 5 to evaluate the performance of the proposed estimator. A real-life data set is analyzed in Section 6 to illustrate the findings in the paper, and Section 7 ends with some concluding remarks.

## 2. A New Estimator

The presence of outliers in the *y*-direction affects the performance of the MRT estimator. Therefore, we suggest a ridge-type modified M-estimator (RTMME). This is defined as

(7) $$\begin{array}{c} {\hat{\beta }}_{\text{RTMME}}\left(k,d\right)=R_{kd}{\hat{\beta }}_{M}, \end{array}$$

where *k* > 0,  0 < *d* < 1. It appears that ${\hat{\beta \;}}_{\text{RTMME}}\left(k,d\right)$ is a general estimator, which includes ${\hat{\beta \;}}_{M}$ and ${\hat{\beta \;}}_{M}\left(k\right)$:

(8) $$\begin{array}{c} {\hat{\beta }}_{\text{RTMME}}\left(0,0\right)={\hat{\beta }}_{M},\\ {\hat{\beta }}_{\text{RTMME}}\left(k,0\right)={\hat{\beta }}_{M}\left(k\right). \end{array}$$

The canonical form of model (1) is written as

(9) $$\begin{array}{c} y=z\alpha +\varepsilon , \end{array}$$

where  *Z*=*XT*,  *α*=*T*′*β*, and *T* is the orthogonal matrix whose columns contain the eigenvectors of *X*′*X*. Then,

(10) $$\begin{array}{c} Z^{\prime }Z=T^{\prime }X^{\prime }XT=\Lambda =\text{diag}\left(\lambda_{1},\lambda_{2},\ldots ,\lambda_{p}\right), \end{array}$$

where  *λ*_1_,  *λ*_2_,  …,  *λ*_*p*_ > 0 are the ordered eigenvalues of *X*′*X*.

Let ${\hat{\alpha }}_{M}$ be M-defined by the solution of the M-estimating equations ∑*φ*(*e*_*i*_/*s*)*z*_*i*_=0, where $e_{i}=y_{i}-z_{i}{\hat{\alpha }}_{M},\;s$ is an estimator of scale for the errors and *φ*(·) is some suitably chosen function [14]. The estimators presented in equations (2)–(7) can be written in canonical form as follows:

(11) $$\begin{array}{c} \hat{\alpha }=\Lambda^{-1}Z^{\prime }y,\\ \hat{\alpha }\left(k\right)={\left(\Lambda +\text{kI}\right)}^{-1}Z^{\prime }y=R_{k}^{*}\hat{\alpha },\\ {\hat{\alpha }}_{M}\left(k\right)=R_{k}^{*}{\hat{\alpha }}_{M},\\ {\hat{\alpha }}_{\text{MRT}}\left(k,d\right)={\left(\Lambda +k\left(1+d\right)I\right)}^{-1}Z^{\prime }y,\\ {\hat{\alpha }}_{\text{MRT}}\left(k,d\right)=R_{kd}\hat{\alpha },\\ {\hat{\alpha }}_{\text{RTMME}}\left(k,d\right)=R_{kd}{\hat{\alpha }}_{M}, \end{array}$$

where *R*_*kd*_=Λ(Λ+*k*(1+*d*)*I*)^−1^, *R*_*k*_^*∗*^=Λ(Λ+*kI*)^−1^, and *k* > 0.

## 3. Superiority of the New Estimator

The mean square error (MSE) criterion is used to compare the performance of the estimators. The following conditions are imposed to present our main theorems:

- *φ* is skew-symmetric and nondecreasing
- The errors are symmetric
- Ω  is finite

Note that any estimator of *α* has a corresponding relation $\tilde{\beta }=\;T^{\prime }\tilde{\alpha }$ such that $\text{MSE}\left(\tilde{\beta }\right)=\text{MSE}\left(\tilde{\alpha }\right)$. Thus, it is sufficient to consider the canonical form only. The MSEs of the aforementioned estimators are derived to be

(12) $$\begin{array}{c} \text{MSE}\left(\hat{\alpha }\right)=\sigma^{2}\sum_{i=1}^{p}\frac{1}{\lambda_{i}},\\ \text{MSE}\left({\hat{\alpha }}_{M}\right)=\sum_{i=1}^{p}\Omega_{ii},\\ \text{MSE}\left[\hat{\alpha }\left(k\right)\right]=\sigma^{2}\sum_{i=1}^{p}\frac{\lambda_{i}}{{\left(\lambda_{i}+k\right)}^{2}}+\sum_{i=1}^{p}\frac{k^{2}\alpha_{i}^{2}}{{\left(\lambda_{i}+k\right)}^{2}},\\ \text{MSE}\left[{\hat{\alpha }}_{M}\left(k\right)\right]=\sum_{i=1}^{p}\frac{\lambda_{i}^{2}}{{\left(\lambda_{i}+k\right)}^{2}}\Omega_{ii}+\sum_{i=1}^{p}\frac{k^{2}\alpha_{i}^{2}}{{\left(\lambda_{i}+k\right)}^{2}},\\ \text{MSE}\left[{\hat{\alpha }}_{\text{MRT}}\left(k,d\right)\right]=\sigma^{2}\sum_{i=1}^{p}\frac{\lambda_{i}}{{\left(\lambda_{i}+k\left(1+d\right)\right)}^{2}}+\sum_{i=1}^{p}\frac{k^{2}{\left(1+d\right)}^{2}\alpha_{i}^{2}}{{\left(\lambda_{i}+k\left(1+d\right)\right)}^{2}},\\ \text{MSE}\left[{\hat{\alpha }}_{\text{RTMME}}\left(k,d\right)\right]=\sum_{i=1}^{p}\frac{\lambda_{i}{}^{2}}{{\left(\lambda_{i}+k\left(1+d\right)\right)}^{2}}\Omega_{ii}+\sum_{i=1}^{p}\frac{k^{2}{\left(1+d\right)}^{2}\alpha_{i}^{2}}{{\left(\lambda_{i}+k\left(1+d\right)\right)}^{2}}, \end{array}$$

where $\Omega_{ii}=\text{Cov}\left({\hat{\alpha }}_{M}\right)$.

**Theorem 1 .**

For$\;\text{MSE}\left({\hat{\alpha }}_{\text{RTMME}}\left(k,d\right)\right)<\text{MSE}\left({\hat{\alpha }}_{\text{MRT}}\left(k,\;d\right)\right)$, then ∑_*i*=1_^*p*^*Ω*_*ii*_ < ∑_*i*=1_^*p*^*σ*^2^*λ*_*i*_^−1^ for every *k* > 0 and *i* *=* 1, 2,…, *p*, where Ω_*ii*_ are the diagonal elements of Ω.

**Proof**

After some algebraic manipulation, the difference between $\text{MSE}\left({\hat{\alpha }}_{\text{RTMME}}\left(k,d\right)\right)-\text{MSE}\left({\hat{\alpha }}_{\text{MRT}}\left(k,\;d\right)\right)$ gives

(13) $$\begin{array}{c} \Delta_{1}=\text{MSE}\left({\hat{\alpha }}_{\text{RTMME}}\left(k,d\right)\right)-\text{MSE}\left({\hat{\alpha }}_{MRT}\left(k,d\right)\right), \end{array}$$

(14) $$\begin{array}{c} \Delta_{1}=\sum_{i=1}^{p}\frac{\lambda_{i}{}^{2}\Omega_{ii}-\sigma^{2}\lambda_{i}}{{\left(\lambda_{i}+k\left(1+d\right)\right)}^{2}}. \end{array}$$

For equation (14) to be less than zero, we should have  *Ω*_*ii*_ − *σ*^2^*λ*_*i*_^−1^ < 0, which also implies ∑_*i*=1_^*p*^*Ω*_*ii*_ < ∑_*i*=1_^*p*^*σ*^2^*λ*_*i*_^−1^ for  *k* > 0,  0 < *d* < 1  and for *i* = 1, 2,…, *p*.

**Theorem 2 .**

When $\;\text{MSE}\left({\hat{\alpha }}_{\text{RTMME}}\left(k,d\right)\right)<\text{MSE}\left({\hat{\alpha }}_{M}\left(k\right)\right)$, there exists a positive constant *k* > *k*_*i*_ > 0, where

(15) $$\begin{array}{c} k_{1i}=\frac{\lambda_{i}\left[\sqrt{{\left(\left(d+2\right)\left(\alpha_{i}^{2}-\Omega_{ii}\right)\right)}^{2}+8\alpha_{i}^{2}\Omega_{ii}\left(d+1\right)}-\left(d+2\right)\left(\alpha_{i}^{2}-\Omega_{ii}\right)\right]}{4\alpha_{i}^{2}\left(d+1\right)}. \end{array}$$

**Proof**

$\text{The difference MSE}\left({\hat{\alpha }}_{\text{RTMME}}\left(k,d\right)\right)-\text{MSE}\left({\hat{\alpha }}_{M}\left(k\right)\right)\text{ is}\;:$

(16) $$\begin{array}{c} \Delta_{2}=\sum_{i=1}^{p}\frac{\lambda_{i}{}^{2}\Omega_{ii}+k^{2}{\left(1+d\right)}^{2}\alpha_{i}^{2}}{{\left(\lambda_{i}+k\left(1+d\right)\right)}^{2}}-\sum_{i=1}^{p}\frac{\lambda_{i}{}^{2}\Omega_{ii}+k^{2}\alpha_{i}^{2}}{{\left(\lambda_{i}+k\right)}^{2}}. \end{array}$$

The difference is strictly less than zero if and only if, after simplification, the following expression holds:

(17) $$\begin{array}{c} k^{2}\left[2\alpha_{i}^{2}\lambda_{i}d\right]+k\left[\lambda_{i}{}^{2}d\left(d+2\right)\left(\alpha_{i}^{2}-\Omega_{ii}\right)\right]-2\Omega_{ii}\lambda_{i}{}^{3}d<0. \end{array}$$

Solving inequality (17) for *k*, we get

(18) $$\begin{array}{c} k_{1i}=\frac{\lambda_{i}\left[\sqrt{{\left(\left(d+2\right)\left(\alpha_{i}^{2}-\Omega_{ii}\right)\right)}^{2}+8\alpha_{i}^{2}\Omega_{ii}\left(d+1\right)}-\left(d+2\right)\left(\alpha_{i}^{2}-\Omega_{ii}\right)\right]}{4\alpha_{i}^{2}\left(d+1\right)}. \end{array}$$

Notice that if $\;\lambda_{i}\left[\sqrt{{\left(\left(d+2\right)\left(\;\alpha_{i}^{2}-\Omega_{ii}\right)\right)}^{2}+8\alpha_{i}^{2}\Omega_{ii}\left(d+1\right)}-\;\left(d+2\right)\left(\;\alpha_{i}^{2}-\Omega_{ii}\right)\right]>0\;;\;k_{1i}>0\;$, and there is, therefore, a positive constant  *k* > *k*_1*i*_ > 0.

**Theorem 3 .**

A necessary condition for $\;\text{MSE}\left({\hat{\alpha }}_{\text{RTMME}}\left(k,d\right)\right)<\;\text{MSE}\left({\hat{\alpha }}_{M}\right)$ is

(19) $$\begin{array}{c} \sum_{i=1}^{p}\Omega_{ii}>\sum_{i=1}^{p}\frac{k\left(1+d\right)\alpha_{i}^{2}}{2\lambda_{i}+k\left(1+d\right)}. \end{array}$$

**Proof**

$\text{The difference MSE}\left({\hat{\alpha }}_{\text{RTMME}}\left(k,d\right)\right)-\;\text{MSE}\left({\hat{\alpha }}_{M}\right)\;\text{is}$

(20) $$\begin{array}{c} \Delta_{3}=\sum_{i=1}^{p}\frac{\lambda_{i}{}^{2}\Omega_{ii}+k^{2}{\left(1+d\right)}^{2}\alpha_{i}^{2}}{{\left(\lambda_{i}+k\left(1+d\right)\right)}^{2}}-\sum_{i=1}^{p}\Omega_{ii}, \end{array}$$

(21) $$\begin{array}{c} \Delta_{3}=-\Omega_{ii}\left(2\lambda_{i}k\left(1+d\right)+k^{2}{\left(1+d\right)}^{2}\right)+k^{2}{\left(1+d\right)}^{2}\alpha_{i}^{2}. \end{array}$$

To obtain $\;\text{MSE}\left({\hat{\alpha }}_{\text{RTMME}}\left(k,d\right)\right)<\;\text{MSE}\left({\hat{\alpha }}_{M}\right)$,

(22) $$\begin{array}{c} \sum_{i=1}^{p}\Omega_{ii}<\sum_{i=1}^{p}\frac{k\left(1+d\right)\alpha_{i}^{2}}{2\lambda_{i}+k\left(1+d\right)}. \end{array}$$

Theorem 2 provided *k* > *k*_1*i*_ such that $\text{MSE}\left({\hat{\alpha }}_{\text{RTMME}}\left(k,d\right)\right)<\text{MSE}\left({\hat{\alpha }}_{M}\right)$. Besides this, in the Theorem of Silvapulle [10] (part (i), pg. 321), it is indicated that there exists *k* > 0 such that $\text{MSE}\left({\hat{\alpha }}_{M}\left(k\right)\right)-\text{MSE}\left({\hat{\alpha }}_{M}\right)<0$. Thus, we obtain the corollary as follows.

**Corollary 1 .**

There exists *k* > 0 such that$\;MSE\left({\hat{\alpha }}_{\text{RTMME}}\left(k,d\right)\right)-MSE\left({\hat{\alpha }}_{M}\right)$.

## 4. Robust Choice of *k* and *d* for ${\hat{\alpha }}_{\text{RTMME}}\left(k,d\right)$

For the robust biasing parameters *k* and *d* for the modified two-parameter estimator, the optimal values can be determined by minimizing equation (23) with respect to each of the parameters:

(23) $$\begin{array}{c} f\left(k,d\right)=\sum_{i=1}^{p}\frac{\lambda_{i}{}^{2}}{{\left(\lambda_{i}+k\left(1+d\right)\right)}^{2}}\Omega_{ii}+\sum_{i=1}^{p}\frac{k^{2}{\left(1+d\right)}^{2}\alpha_{i}^{2}}{{\left(\lambda_{i}+k\left(1+d\right)\right)}^{2}}. \end{array}$$

This can be obtained by solving

(24) $$\begin{array}{c} \frac{\partial f\left(k,d\right)}{\partial d}=0,\\ \frac{\partial f\left(k,d\right)}{\partial k}=0. \end{array}$$

By doing this, we have

(25) $$\begin{array}{c} d=\frac{\lambda_{i}\Omega_{ii}}{k\alpha_{i}^{2}}-1, \end{array}$$

(26) $$\begin{array}{c} k=\frac{\lambda_{i}\Omega_{ii}}{\alpha_{i}^{2}\left(1+d\right)}. \end{array}$$

We substitute *Ω*_*ii*_ and *α*_*i*_^2^ into equations (24) and (25) with their corresponding estimates. We assume that ${\hat{\alpha }}_{M}$ is normally distributed with mean *α* and covariance matrix *A*^2^Λ^−1^. This assumption holds since $n^{1/2}\left({\hat{\alpha }}_{M}^{2}-\alpha \right)⟶N\left(0,A^{2}\Lambda^{-1}\right)$, where

(27) $$\begin{array}{c} A^{2}=\frac{s_{o}^{2}E\left(\varphi^{2}\left(\varepsilon /s_{o}\right)\right)}{{\left(E\left(\varphi^{\prime }\left(\varepsilon /s_{o}\right)\right)\right)}^{2}}, \end{array}$$

with the scale estimate *s*_*o*_. Thus, the estimate of $\alpha_{i}^{2}\text{ is }{\hat{\alpha }}_{Mi}^{2}$, and the unbiased estimator of *Ω*_*ii*_ is asymptotically ${\hat{A}}^{2}/\lambda_{i}$, where ${\hat{A}}^{2}$ is given by Huber [9] as

(28) $$\begin{array}{c} {\hat{A}}^{2}=\frac{s^{2}{\left(n-p\right)}^{-1}\sum_{i=1}^{p}{\left(\varphi \left(e_{i}/s\right)\right)}^{2}}{{\left(\sum_{i=1}^{p}\left(1/n\right)\varphi^{\prime }\left(e_{i}/s\right)\right)}^{2}}. \end{array}$$

We get the optimal estimator of *d* and *k* as

(29) $$\begin{array}{c} d=\frac{{\hat{A}}^{2}}{k{\hat{\alpha }}_{Mi}^{2}}-1,\;i=1,2,\ldots ,p, \end{array}$$

(30) $$\begin{array}{c} k=\frac{{\hat{A}}^{2}}{\left(1+d\right){\hat{\alpha }}_{Mi}^{2}},\;i=1,2,\ldots ,p. \end{array}$$

Following Kibria [15], the arithmetic and geometric mean version of *k* is obtained, respectively, as

(31) $$\begin{array}{c} {\tilde{k}}_{\text{GMR}}={\left[\prod_{i=1}^{p}\frac{{\hat{A}}^{2}}{\left(1+d\right){\hat{\alpha }}_{Mi}^{2}}\right]}^{1/p}, \end{array}$$

(32) $$\begin{array}{c} {\tilde{k}}_{\text{AMR}}=\frac{1}{p}\sum_{i=1}^{p}\frac{{\hat{A}}^{2}}{\left(1+d\right){\hat{\alpha }}_{Mi}^{2}} \end{array}$$

The harmonic mean version is generally preferred to other versions [3]. Hence, the robust harmonic mean version of the proposed *d* and *k* from (31) and (32) is obtained as

(33) $$\begin{array}{c} {\tilde{k}}_{\text{HMR}}=\frac{p{\hat{A}}^{2}}{\sum_{i=1}^{p}\left(1+d\right){\hat{\alpha }}_{Mi}^{2}}, \end{array}$$

(34) $$\begin{array}{c} {\tilde{d}}_{\text{HMR}}=\frac{p}{\sum_{i=1}^{p}\left(1/d\right)}. \end{array}$$

The selection of the estimators of the parameters *d* and *k* can be obtained iteratively as follows:

  Step 1: use $\hat{d}=\min\left({\hat{A}}^{2}/{\hat{\alpha }}_{Mi}^{2}\right)$ to obtain an initial estimate for *d*
  Step 2: from (33), get ${\tilde{k}}_{\text{HMR}}$ using *d* in Step 1
  Step 3: calculate ${\tilde{d}}_{\text{HMR}}$ in (34) by using ${\tilde{k}}_{\text{HMR}}$ in Step 2
  Step 4: use $\hat{d}$ in Step 1 if ${\tilde{d}}_{\text{HMR}}$ < 0

## 5. Monte Carlo Simulation Study

We adopted the simulation design by McDonald and Galarneau [16], Kibria [15], and Lukman et al. [17]. The explanatory variables are generated using the following equation:

(35) $$\begin{array}{c} x_{ij}={\left(1-\rho^{2}\right)}^{1/2}z_{ij}+\rho z_{i,p+1},\;i=1,2,\ldots ,n,j=1,2,\ldots ,p, \end{array}$$

where  *ρ*^2^ denotes the correlation between explanatory variables and *z*_*ij*_ are pseudo-random numbers from the standard normal distribution. The coefficients *β*_1_, *β*_2_,…,  *β*_*p*_ are selected as the normalized eigenvectors corresponding to the largest eigenvalue of *X*′*X* so that we have  *β*′*β* = 1, which is a common restriction in simulation studies of this type ([3]). The dependent variable is then determined using

(36) $$\begin{array}{c} y_{i}=\beta_{0}+\beta_{1}x_{i1}+\beta_{2}x_{i2}+\cdots +\beta_{p}x_{ip}+\varepsilon_{i},\;i=1,2,\ldots ,n, \end{array}$$

where the error term *ε*_*i*_′*s* is generated with mean and variance 0 and *σ*^2^, respectively. We fixed the number of explanatory variables to three and seven (*p* = 3, 7), and other parameters such as *ρ*, *σ*, and *n* were varied; their values considered in this study are given as follows:

 
*ρ* = 0.7,  0.8,  0.9 and 0.99
  The standard deviation (*σ*) of the error term in this simulation study is 1,  5,  10
 
*n* = 20,  50,  100

We considered three different cases in this study:

  Case I: no outlier
  Case II: one outlier
  Case III: two outliers

In the case of no outliers, equation (36) is taken into consideration. For the case of one outlier, the tenth observation is changed as *y*_10_^*∗*^=*y*_10_+20*σ*. For the case of two outliers, the fifth and the tenth observations are changed as *y*_5_^*∗*^=*y*_5_+20*σ*  and *y*_10_^*∗*^=*y*_10_ −  20*σ*, respectively. The experiment is replicated 2,000 times by generating new pseudo-random numbers, and the estimated MSE is calculated as

(37) $$\begin{array}{c} \text{mse}\left(\hat{\alpha }\right)=\frac{1}{2000}\sum_{j=1}^{2000}{\left({\hat{\alpha }}_{ij}-\alpha_{i}\right)}^{\prime }\left({\hat{\alpha }}_{ij}-\alpha_{i}\right). \end{array}$$

The results of the simulation are presented in Tables 1–18. As expected, the OLSE is observed to have the least performance. The following observations are also made:

- As the error standard deviation (*σ*) and the degree of multicollinearity (*ρ*) increase, the MSEs of the estimators ($\alpha ,{\hat{\alpha }}_{M},\hat{\alpha }\left(k\right),\;{\hat{\alpha }}_{M}\left(k\right),\;{\hat{\alpha }}_{\text{MRT}},\text{ and }{\hat{\alpha }}_{\text{RTMME}}$) increase.
- As the biasing parameters *k* and *d* increase, the MSEs of the estimators ($\alpha ,{\hat{\alpha }}_{M},\hat{\alpha }\left(k\right),\;{\hat{\alpha }}_{M}\left(k\right),\;{\hat{\alpha }}_{\text{MRT}},\;\text{and}\;{\hat{\alpha }}_{\text{RTMME}}$) also decrease.
- The MSEs of the estimators ($\alpha ,{\hat{\alpha }}_{M},\hat{\alpha }\left(k\right),\;{\hat{\alpha }}_{M}\left(k\right),\;{\hat{\alpha }}_{\text{MRT}}$, and ${\hat{\alpha }}_{\text{RTMME}}$) decrease as the sample size increases. However, as the number of outliers increases, the MSEs also increase.
- Finally, just as in the outcome of Lukman et al. [13], the MRT estimator outperforms other estimators considered in the case of no outlier. However, when outliers were introduced, the RTMME outperforms other considered estimators.

## 6. Numerical Example

Hussein data were originally adopted by Hussein and Zari [18] and later by Lukman et al. [19, 20]. The data contain 31 observations and three explanatory variables. The variance inflation factor for the three explanatory variables is greater than 10 (VIF> 10), which indicates multicollinearity. Lukman et al. [20] identified the following observations as outliers in the *y*-direction: 12, 14, 15, 16, 30, and 31. Hence, this data set suffers both problems considered in this study.  Table 19 shows that the estimated MSE value of the new estimator, RTMME, is smaller compared to the ridge, M-ridge, and MRT estimator.

The theoretical results in this study are validated through the Hussein data as follows:

- ∑_*i*=1_^*p*^*Ω*_*ii*_=883.55 < ∑_*i*=1_^*p*^*σ*^2^*λ*_*i*_^−1^=1850.482; thus, $\text{MSE}\left({\hat{\alpha }}_{\text{RTMME}}\left(k,d\right)\right)<\text{MSE}\left({\hat{\alpha }}_{\text{MRT}}\left(k,\;d\right)\right)$ for every *k* > 0. By Theorem 1, this implies that the RTMME outperforms the MRT estimator.
- From Theorem 2, k_1i_ is calculated as [0.2470, 0.0520, 0.0519, 0.0002]. For *k* > 1.2980 (*k*> *k*_1*i*_), $\text{MSE}\left({\hat{\alpha }}_{\text{RTMME}}\left(k,d\right)\right)<\text{MSE}\left({\hat{\alpha }}_{M}\left(k\right)\right).$
- The necessary condition from Theorem 3 is also proven from  ∑_*i*=1_^*p*^*Ω*_*ii*_ < ∑_*i*=1_^*p*^∑_*i*=1_^*p*^(*k*(1+*d*)*α*_*i*_^2^)/(2*λ*_*i*_+*k*(1+*d*))=3026.59 =>$\text{MSE}\left({\hat{\alpha }}_{\text{RTMME}}\left(k,d\right)\right)<\text{MSE}\left({\hat{\alpha }}_{M}\right)$.

## 7. Conclusion

We proposed a two-parameter ridge-type modified M-estimator to jointly handle the problem of multicollinearity and outliers in a linear regression model. Theoretically, the new estimator outperforms the existing estimators under certain conditions. The results of the simulation study and numerical example agree with the theoretical findings. A right choice of *k* and *d* also produces better estimates using the proposed estimator. Thus, in the presence of multicollinearity and outliers, this estimator can effectively replace the following estimators: the ordinary least squares estimator, the M-estimator, the ridge estimator, the M-ridge estimator, and the modified ridge-type estimator.

## Figures and Tables

**Table 1:** Estimated MSEs of OLS, MRE, MRE, MRT, and RTMME at *n* = 20 and *p* = 3 when there is no outlier.

*k*	Sigma	Rho	*d* = 0.2						*d* = 0.5						*d* = 0.8					
			$\hat{\alpha }$	${\hat{\alpha }}_{M}$	$\hat{\alpha }\left(k\right)$	${\hat{\alpha }}_{M}\left(k\right)$	${\hat{\alpha }}_{\text{MRT}}$	${\hat{\alpha }}_{\text{RTMME}}$	$\hat{\alpha }$	${\hat{\alpha }}_{M}$	$\hat{\alpha }\left(k\right)$	${\hat{\alpha }}_{M}\left(k\right)$	${\hat{\alpha }}_{\text{MRT}}$	${\hat{\alpha }}_{\text{RTMME}}$	$\hat{\alpha }$	${\hat{\alpha }}_{M}$	$\hat{\alpha }\left(k\right)$	${\hat{\alpha }}_{M}\left(k\right)$	${\hat{\alpha }}_{\text{MRT}}$	${\hat{\alpha }}_{\text{RTMME}}$
0.3	1	0.7	8.973	4.898	8.208	4.483	8.069	4.408	8.973	4.898	8.208	4.483	7.867	4.299	8.973	4.898	8.208	4.483	7.675	4.195
		0.8	13.374	7.518	11.664	6.544	11.366	6.375	13.374	7.518	11.664	6.544	10.943	6.135	13.374	7.518	11.664	6.544	10.547	5.911
		0.9	0.945	0.980	0.930	0.966	0.681	0.707	0.945	0.980	0.930	0.966	0.635	0.659	0.945	0.980	0.930	0.966	0.595	0.618
		0.99	11.197	11.806	9.351	10.000	1.344	1.404	11.197	11.806	9.351	10.000	1.050	1.094	11.197	11.806	9.351	10.000	0.858	0.892
	5	0.7	0.789	0.623	0.785	0.620	0.784	0.619	0.789	0.623	0.785	0.620	0.783	0.618	0.789	0.623	0.785	0.620	0.782	0.617
		0.8	1.161	0.893	1.152	0.886	1.151	0.884	1.161	0.893	1.152	0.886	1.148	0.882	1.161	0.893	1.152	0.886	1.145	0.880
		0.9	0.945	0.980	0.944	0.979	0.931	0.965	0.945	0.980	0.944	0.979	0.927	0.962	0.945	0.980	0.944	0.979	0.924	0.958
		0.99	11.197	11.806	11.112	11.723	9.377	9.882	11.197	11.806	11.112	11.723	8.998	9.481	11.197	11.806	11.112	11.723	8.644	9.107
	10	0.7	0.179	0.115	0.179	0.115	0.179	0.115	0.179	0.115	0.179	0.115	0.179	0.114	0.179	0.115	0.179	0.115	0.179	0.114
		0.8	0.259	0.170	0.259	0.170	0.259	0.170	0.259	0.170	0.259	0.170	0.258	0.170	0.259	0.170	0.259	0.170	0.258	0.170
		0.9	0.945	0.980	0.944	0.980	0.941	0.976	0.945	0.980	0.944	0.980	0.940	0.975	0.945	0.980	0.944	0.980	0.939	0.974
		0.99	11.197	11.806	11.176	11.785	10.690	11.270	11.197	11.806	11.176	11.785	10.569	11.142	11.197	11.806	11.176	11.785	10.450	11.016

0.7	1	0.7	8.973	4.898	7.352	4.020	7.089	3.878	8.973	4.898	7.352	4.020	6.724	3.682	8.973	4.898	7.352	4.020	6.391	3.503
		0.8	13.374	7.518	9.900	5.545	9.388	5.256	13.374	7.518	9.900	5.545	8.700	4.869	13.374	7.518	9.900	5.545	8.095	4.529
		0.9	0.945	0.980	0.930	0.966	0.493	0.511	0.945	0.980	0.930	0.966	0.441	0.457	0.945	0.980	0.930	0.966	0.400	0.415
		0.99	11.197	11.806	9.351	10.000	0.542	0.559	11.197	11.806	9.351	10.000	0.444	0.455	11.197	11.806	9.351	10.000	0.386	0.395
	5	0.7	0.789	0.623	0.780	0.616	0.778	0.614	0.789	0.623	0.780	0.616	0.775	0.612	0.789	0.623	0.780	0.616	0.772	0.610
		0.8	1.161	0.893	1.141	0.876	1.137	0.873	1.161	0.893	1.141	0.876	1.131	0.868	1.161	0.893	1.141	0.876	1.125	0.864
		0.9	0.945	0.980	0.944	0.979	0.912	0.947	0.945	0.980	0.944	0.979	0.905	0.939	0.945	0.980	0.944	0.979	0.897	0.931
		0.99	11.197	11.806	11.112	11.723	7.617	8.022	11.197	11.806	11.112	11.723	7.013	7.384	11.197	11.806	11.112	11.723	6.484	6.826
	10	0.7	0.179	0.115	0.178	0.114	0.178	0.114	0.179	0.115	0.178	0.114	0.178	0.114	0.179	0.115	0.178	0.114	0.178	0.114
		0.8	0.259	0.170	0.258	0.170	0.258	0.169	0.259	0.170	0.258	0.170	0.257	0.169	0.259	0.170	0.258	0.170	0.257	0.169
		0.9	0.945	0.980	0.944	0.980	0.936	0.971	0.945	0.980	0.944	0.980	0.934	0.969	0.945	0.980	0.944	0.980	0.932	0.967
		0.99	11.197	11.806	11.176	11.785	10.071	10.615	11.197	11.806	11.176	11.785	9.818	10.348	11.197	11.806	11.176	11.785	9.576	10.093

0.9	1	0.7	8.973	4.898	6.981	3.820	6.674	3.655	8.973	4.898	6.981	3.820	6.257	3.431	8.973	4.898	6.981	3.820	5.885	3.232
		0.8	13.374	7.518	9.182	5.140	8.609	4.817	13.374	7.518	9.182	5.140	7.858	4.396	13.374	7.518	9.182	5.140	7.213	4.035
		0.9	0.945	0.980	0.930	0.966	0.434	0.450	0.945	0.980	0.930	0.966	0.385	0.399	0.945	0.980	0.930	0.966	0.348	0.361
		0.99	11.197	11.806	9.351	10.000	0.434	0.445	11.197	11.806	9.351	10.000	0.368	0.376	11.197	11.806	9.351	10.000	0.331	0.337
	5	0.7	0.789	0.623	0.777	0.614	0.775	0.612	0.789	0.623	0.777	0.614	0.771	0.609	0.789	0.623	0.777	0.614	0.767	0.606
		0.8	1.161	0.893	1.135	0.872	1.130	0.868	1.161	0.893	1.135	0.872	1.122	0.862	1.161	0.893	1.135	0.872	1.115	0.856
		0.9	0.945	0.980	0.944	0.979	0.904	0.938	0.945	0.980	0.944	0.979	0.894	0.927	0.945	0.980	0.944	0.979	0.884	0.918
		0.99	11.197	11.806	11.112	11.723	6.933	7.299	11.197	11.806	11.112	11.723	6.278	6.608	11.197	11.806	11.112	11.723	5.720	6.019
	10	0.7	0.179	0.115	0.178	0.114	0.178	0.114	0.179	0.115	0.178	0.114	0.178	0.114	0.179	0.115	0.178	0.114	0.178	0.114
		0.8	0.259	0.170	0.258	0.169	0.257	0.169	0.259	0.170	0.258	0.169	0.257	0.169	0.259	0.170	0.258	0.169	0.257	0.169
		0.9	0.945	0.980	0.944	0.980	0.934	0.969	0.945	0.980	0.944	0.980	0.931	0.966	0.945	0.980	0.944	0.980	0.929	0.964
		0.99	11.197	11.806	11.176	11.785	9.783	10.311	11.197	11.806	11.176	11.785	9.476	9.986	11.197	11.806	11.176	11.785	9.184	9.678

**Table 2:** Estimated MSEs of OLS, MRE, MRE, MRT, and RTMME at *n* = 20 and *p* = 3 when there is one outlier.

*k*	Sigma	Rho	*d* = 0.2						*d* = 0.5						*d* = 0.8					
			$\hat{\alpha }$	${\hat{\alpha }}_{M}$	$\hat{\alpha }\left(k\right)$	${\hat{\alpha }}_{M}\left(k\right)$	${\hat{\alpha }}_{\text{MRT}}$	${\hat{\alpha }}_{\text{RTMME}}$	$\hat{\alpha }$	${\hat{\alpha }}_{M}$	$\hat{\alpha }\left(k\right)$	${\hat{\alpha }}_{M}\left(k\right)$	${\hat{\alpha }}_{\text{MRT}}$	${\hat{\alpha }}_{\text{RTMME}}$	$\hat{\alpha }$	${\hat{\alpha }}_{M}$	$\hat{\alpha }\left(k\right)$	${\hat{\alpha }}_{M}\left(k\right)$	${\hat{\alpha }}_{\text{MRT}}$	${\hat{\alpha }}_{\text{RTMME}}$
0.3	1	0.7	14.015	6.603	12.842	6.066	12.628	5.968	14.015	6.603	12.842	6.066	12.319	5.827	14.015	6.603	12.842	6.066	12.023	5.691
		0.8	21.072	9.888	18.352	8.633	17.879	8.414	21.072	9.888	18.352	8.633	17.207	8.104	21.072	9.888	18.352	8.633	16.576	7.813
		0.9	608.818	1.152	592.046	1.140	410.694	0.853	608.818	1.152	592.046	1.140	376.593	0.800	608.818	1.152	592.046	1.140	346.658	0.753
		0.99	8184.099	14.132	5874.695	12.202	574.320	1.601	8184.099	14.132	5874.695	12.202	411.088	1.239	8184.099	14.132	5874.695	12.202	308.936	1.004
	5	0.7	1.125	0.924	1.120	0.920	1.119	0.919	1.125	0.924	1.120	0.920	1.118	0.918	1.125	0.924	1.120	0.920	1.116	0.917
		0.8	1.639	1.292	1.628	1.284	1.626	1.282	1.639	1.292	1.628	1.284	1.623	1.279	1.639	1.292	1.628	1.284	1.620	1.277
		0.9	122.561	1.152	122.425	1.152	120.476	1.136	122.561	1.152	122.425	1.152	119.964	1.133	122.561	1.152	122.425	1.152	119.454	1.129
		0.99	1646.049	14.129	1622.592	14.043	1331.117	11.784	1646.049	14.129	1622.592	14.043	1266.599	11.296	1646.049	14.129	1622.592	14.043	1206.688	10.841
	10	0.7	0.203	0.127	0.203	0.127	0.203	0.127	0.203	0.127	0.203	0.127	0.203	0.127	0.203	0.127	0.203	0.127	0.203	0.127
		0.8	0.306	0.190	0.306	0.190	0.306	0.190	0.306	0.190	0.306	0.190	0.305	0.190	0.306	0.190	0.306	0.190	0.305	0.190
		0.9	61.768	1.152	61.751	1.152	61.506	1.148	61.768	1.152	61.751	1.152	61.441	1.147	61.768	1.152	61.751	1.152	61.376	1.146
		0.99	828.724	14.128	825.755	14.106	784.255	13.474	828.724	14.128	825.755	14.106	773.701	13.318	828.724	14.128	825.755	14.106	763.361	13.165

0.7	1	0.7	14.015	6.603	11.526	5.464	11.120	5.279	14.015	6.603	11.526	5.464	10.558	5.022	14.015	6.603	11.526	5.464	10.044	4.787
		0.8	21.072	9.888	15.549	7.339	14.735	6.963	21.072	9.888	15.549	7.339	13.644	6.459	21.072	9.888	15.549	7.339	12.684	6.016
		0.9	608.818	1.152	592.046	1.140	269.408	0.631	608.818	1.152	592.046	1.140	230.019	0.568	608.818	1.152	592.046	1.140	198.852	0.517
		0.99	8184.099	14.132	5874.695	12.202	148.737	0.616	8184.099	14.132	5874.695	12.202	101.143	0.496	8184.099	14.132	5874.695	12.202	73.364	0.425
	5	0.7	1.125	0.924	1.114	0.914	1.112	0.912	1.125	0.924	1.114	0.914	1.108	0.910	1.125	0.924	1.114	0.914	1.105	0.907
		0.8	1.639	1.292	1.614	1.272	1.610	1.268	1.639	1.292	1.614	1.272	1.603	1.262	1.639	1.292	1.614	1.272	1.596	1.256
		0.9	122.561	1.152	122.425	1.152	117.781	1.116	122.561	1.152	122.425	1.152	116.631	1.108	122.561	1.152	122.425	1.152	115.498	1.099
		0.99	1646.049	14.129	1622.592	14.043	1035.101	9.523	1646.049	14.129	1622.592	14.043	935.995	8.750	1646.049	14.129	1622.592	14.043	850.550	8.075
	10	0.7	0.203	0.127	0.203	0.127	0.203	0.127	0.203	0.127	0.203	0.127	0.203	0.127	0.203	0.127	0.203	0.127	0.202	0.127
		0.8	0.306	0.190	0.305	0.190	0.305	0.190	0.306	0.190	0.305	0.190	0.304	0.189	0.306	0.190	0.305	0.190	0.304	0.189
		0.9	61.768	1.152	61.751	1.152	61.160	1.143	61.768	1.152	61.751	1.152	61.009	1.141	61.768	1.152	61.751	1.152	60.859	1.138
		0.99	828.724	14.128	825.755	14.106	730.360	12.676	828.724	14.128	825.755	14.106	708.525	12.351	828.724	14.128	825.755	14.106	687.663	12.040

0.9	1	0.7	14.015	6.603	10.954	5.203	10.481	4.987	14.015	6.603	10.954	5.203	9.837	4.692	14.015	6.603	10.954	5.203	9.260	4.429
		0.8	21.072	9.888	14.408	6.812	13.499	6.392	21.072	9.888	14.408	6.812	12.307	5.842	21.072	9.888	14.408	6.812	11.285	5.370
		0.9	608.818	1.152	592.046	1.140	225.122	0.560	608.818	1.152	592.046	1.140	187.451	0.499	608.818	1.152	592.046	1.140	158.695	0.452
		0.99	8184.099	14.132	5874.695	12.202	96.278	0.484	8184.099	14.132	5874.695	12.202	64.898	0.403	8184.099	14.132	5874.695	12.202	46.836	0.358
	5	0.7	1.125	0.924	1.111	0.912	1.108	0.909	1.125	0.924	1.111	0.912	1.104	0.905	1.125	0.924	1.111	0.912	1.100	0.902
		0.8	1.639	1.292	1.608	1.266	1.602	1.261	1.639	1.292	1.608	1.266	1.593	1.254	1.639	1.292	1.608	1.266	1.584	1.246
		0.9	122.561	1.152	122.425	1.152	116.468	1.106	122.561	1.152	122.425	1.152	115.018	1.095	122.561	1.152	122.425	1.152	113.596	1.085
		0.99	1646.049	14.129	1622.592	14.043	923.015	8.648	1646.049	14.129	1622.592	14.043	817.497	7.812	1646.049	14.129	1622.592	14.043	729.190	7.100
	10	0.7	0.203	0.127	0.203	0.127	0.203	0.127	0.203	0.127	0.203	0.127	0.202	0.127	0.203	0.127	0.203	0.127	0.202	0.126
		0.8	0.306	0.190	0.305	0.190	0.304	0.189	0.306	0.190	0.305	0.190	0.304	0.189	0.306	0.190	0.305	0.190	0.303	0.189
		0.9	61.768	1.152	61.751	1.152	60.988	1.140	61.768	1.152	61.751	1.152	60.795	1.137	61.768	1.152	61.751	1.152	60.603	1.134
		0.99	828.724	14.128	825.755	14.106	705.487	12.306	828.724	14.128	825.755	14.106	679.005	11.910	828.724	14.128	825.755	14.106	653.998	11.535

**Table 3:** Estimated MSEs of OLS, MRE, MRE, MRT, and RTMME at *n* = 20 and *p* = 3 when there are two outliers.

*k*	Sigma	Rho	*d* = 0.2						*d* = 0.5						*d* = 0.8					
			$\hat{\alpha }$	${\hat{\alpha }}_{M}$	$\hat{\alpha }\left(k\right)$	${\hat{\alpha }}_{M}\left(k\right)$	${\hat{\alpha }}_{\text{MRT}}$	${\hat{\alpha }}_{\text{RTMME}}$	$\hat{\alpha }$	${\hat{\alpha }}_{M}$	$\hat{\alpha }\left(k\right)$	${\hat{\alpha }}_{M}\left(k\right)$	${\hat{\alpha }}_{\text{MRT}}$	${\hat{\alpha }}_{\text{RTMME}}$	$\hat{\alpha }$	${\hat{\alpha }}_{M}$	$\hat{\alpha }\left(k\right)$	${\hat{\alpha }}_{M}\left(k\right)$	${\hat{\alpha }}_{\text{MRT}}$	${\hat{\alpha }}_{\text{RTMME}}$
0.3	1	0.7	21.041	16.798	19.134	15.122	18.786	14.819	21.041	16.798	19.134	15.122	18.282	14.381	21.041	16.798	19.134	15.122	17.800	13.964
		0.8	29.866	24.389	26.044	20.913	25.374	20.312	29.866	24.389	26.044	20.913	24.420	19.459	29.866	24.389	26.044	20.913	23.522	18.661
		0.9	875.795	2.113	832.443	2.065	697.950	1.534	875.795	2.113	832.443	2.065	662.429	1.430	875.795	2.113	832.443	2.065	629.686	1.336
		0.99	8864.956	25.822	5151.316	19.581	1588.579	2.684	8864.956	25.822	5151.316	19.581	1227.830	1.984	8864.956	25.822	5151.316	19.581	978.504	1.530
	5	0.7	0.909	0.893	0.904	0.889	0.904	0.888	0.909	0.893	0.904	0.889	0.902	0.886	0.909	0.893	0.904	0.889	0.901	0.885
		0.8	1.276	1.242	1.267	1.233	1.265	1.231	1.276	1.242	1.267	1.233	1.263	1.229	1.276	1.242	1.267	1.233	1.260	1.226
		0.9	176.002	2.111	175.637	2.109	174.288	2.081	176.002	2.111	175.637	2.109	173.864	2.074	176.002	2.111	175.637	2.109	173.441	2.067
		0.99	1779.071	25.812	1732.191	25.503	1575.743	21.596	1779.071	25.812	1732.191	25.503	1531.295	20.716	1779.071	25.812	1732.191	25.503	1489.009	19.893
	10	0.7	0.218	0.146	0.218	0.146	0.218	0.146	0.218	0.146	0.218	0.146	0.218	0.146	0.218	0.146	0.218	0.146	0.218	0.146
		0.8	0.332	0.220	0.332	0.219	0.332	0.219	0.332	0.220	0.332	0.219	0.332	0.219	0.332	0.220	0.332	0.219	0.331	0.219
		0.9	88.506	2.111	88.460	2.110	88.288	2.103	88.506	2.111	88.460	2.110	88.234	2.101	88.506	2.111	88.460	2.110	88.179	2.100
		0.99	894.056	25.809	888.028	25.731	866.223	24.636	894.056	25.809	888.028	25.731	859.509	24.356	894.056	25.809	888.028	25.731	852.888	24.082

0.7	1	0.7	21.041	16.798	16.992	13.267	16.331	12.701	21.041	16.798	16.992	13.267	15.415	11.921	21.041	16.798	16.992	13.267	14.577	11.214
		0.8	29.866	24.389	22.050	17.365	20.879	16.343	29.866	24.389	22.050	17.365	19.299	14.977	29.866	24.389	22.050	17.365	17.899	13.782
		0.9	875.795	2.113	832.443	2.065	537.079	1.089	875.795	2.113	832.443	2.065	484.307	0.958	875.795	2.113	832.443	2.065	439.189	0.851
		0.99	8864.956	25.822	5151.316	19.581	533.881	0.791	8864.956	25.822	5151.316	19.581	383.311	0.569	8864.956	25.822	5151.316	19.581	289.637	0.442
	5	0.7	0.909	0.893	0.899	0.883	0.897	0.881	1.125	0.924	1.114	0.914	1.108	0.910	0.909	0.893	0.899	0.883	0.891	0.875
		0.8	1.276	1.242	1.255	1.221	1.251	1.217	1.639	1.292	1.614	1.272	1.603	1.262	1.276	1.242	1.255	1.221	1.240	1.205
		0.9	176.002	2.111	175.637	2.109	172.045	2.043	176.002	2.111	175.637	2.109	171.078	2.026	176.002	2.111	175.637	2.109	170.119	2.010
		0.99	1779.071	25.812	1732.191	25.503	1361.739	17.499	1779.071	25.812	1732.191	25.503	1283.348	16.089	1779.071	25.812	1732.191	25.503	1212.316	14.853
	10	0.7	0.218	0.146	0.218	0.146	0.218	0.146	0.218	0.146	0.218	0.146	0.218	0.146	0.218	0.146	0.218	0.146	0.218	0.145
		0.8	0.332	0.220	0.331	0.219	0.331	0.219	0.332	0.220	0.331	0.219	0.331	0.219	0.332	0.220	0.331	0.219	0.330	0.218
		0.9	88.506	2.111	88.460	2.110	87.999	2.093	88.506	2.111	88.460	2.110	87.872	2.089	88.506	2.111	88.460	2.110	87.747	2.085
		0.99	894.056	25.809	888.028	25.731	831.465	23.202	894.056	25.809	888.028	25.731	817.035	22.618	894.056	25.809	888.028	25.731	803.045	22.056

0.9	1	0.7	21.041	16.798	16.061	12.470	15.290	11.816	21.041	16.798	16.061	12.470	14.240	10.930	21.041	16.798	16.061	12.470	13.300	10.145
		0.8	29.866	24.389	20.407	15.933	19.089	14.797	29.866	24.389	20.407	15.933	17.348	13.315	29.866	24.389	20.407	15.933	15.845	12.054
		0.9	875.795	2.113	832.443	2.065	477.431	0.941	875.795	2.113	832.443	2.065	421.825	0.812	875.795	2.113	832.443	2.065	375.665	0.711
		0.99	8864.956	25.822	5151.316	19.581	367.250	0.546	8864.956	25.822	5151.316	19.581	260.049	0.404	8864.956	25.822	5151.316	19.581	195.002	0.326
	5	0.7	0.909	0.893	0.896	0.880	0.894	0.877	0.909	0.893	0.896	0.880	0.890	0.874	0.909	0.893	0.896	0.880	0.887	0.870
		0.8	1.276	1.242	1.250	1.215	1.245	1.210	1.276	1.242	1.250	1.215	1.237	1.202	1.276	1.242	1.250	1.215	1.230	1.195
		0.9	176.002	2.111	175.637	2.109	170.940	2.024	176.002	2.111	175.637	2.109	169.711	2.003	176.002	2.111	175.637	2.109	168.496	1.983
		0.99	1779.071	25.812	1732.191	25.503	1272.776	15.902	1779.071	25.812	1732.191	25.503	1183.871	14.370	1779.071	25.812	1732.191	25.503	1104.875	13.061
	10	0.7	0.218	0.146	0.218	0.146	0.218	0.145	0.218	0.146	0.218	0.146	0.218	0.145	0.218	0.146	0.218	0.146	0.217	0.145
		0.8	0.332	0.220	0.331	0.219	0.331	0.219	0.332	0.220	0.331	0.219	0.330	0.218	0.332	0.220	0.331	0.219	0.330	0.218
		0.9	88.506	2.111	88.460	2.110	87.854	2.088	88.506	2.111	88.460	2.110	87.693	2.083	88.506	2.111	88.460	2.110	87.531	2.077
		0.99	894.056	25.809	888.028	25.731	815.010	22.536	894.056	25.809	888.028	25.731	797.179	21.823	894.056	25.809	888.028	25.731	780.028	21.146

**Table 4:** Estimated MSEs of OLS, MRE, MRE, MRT, and RTMME at *n* = 50 and *p* = 3 when there is no outlier.

*k*	Sigma	Rho	*d* = 0.2						*d* = 0.5						*d* = 0.8					
			$\hat{\alpha }$	${\hat{\alpha }}_{M}$	$\hat{\alpha }\left(k\right)$	${\hat{\alpha }}_{M}\left(k\right)$	${\hat{\alpha }}_{\text{MRT}}$	${\hat{\alpha }}_{\text{RTMME}}$	$\hat{\alpha }$	${\hat{\alpha }}_{M}$	$\hat{\alpha }\left(k\right)$	${\hat{\alpha }}_{M}\left(k\right)$	${\hat{\alpha }}_{\text{MRT}}$	${\hat{\alpha }}_{\text{RTMME}}$	$\hat{\alpha }$	${\hat{\alpha }}_{M}$	$\hat{\alpha }\left(k\right)$	${\hat{\alpha }}_{M}\left(k\right)$	${\hat{\alpha }}_{\text{MRT}}$	${\hat{\alpha }}_{\text{RTMME}}$
0.3	1	0.7	4.889	2.753	4.735	2.671	4.706	2.655	4.889	2.753	4.735	2.671	4.662	2.631	4.889	2.753	4.735	2.671	4.619	2.608
		0.8	7.282	4.247	6.928	4.046	6.860	4.008	7.282	4.247	6.928	4.046	6.762	3.952	7.282	4.247	6.928	4.046	6.666	3.897
		0.9	0.339	0.356	0.337	0.354	0.296	0.311	0.339	0.356	0.337	0.354	0.287	0.301	0.339	0.356	0.337	0.354	0.278	0.292
		0.99	4.000	4.247	3.714	3.968	1.247	1.322	4.000	4.247	3.714	3.968	1.046	1.108	4.000	4.247	3.714	3.968	0.898	0.950
	5	0.7	0.242	0.156	0.241	0.156	0.241	0.156	0.242	0.156	0.241	0.156	0.241	0.156	0.242	0.156	0.241	0.156	0.241	0.156
		0.8	0.362	0.233	0.361	0.232	0.361	0.232	0.362	0.233	0.361	0.232	0.361	0.232	0.362	0.233	0.361	0.232	0.361	0.232
		0.9	0.339	0.356	0.339	0.356	0.337	0.354	0.339	0.356	0.339	0.356	0.337	0.354	0.339	0.356	0.339	0.356	0.336	0.353
		0.99	4.000	4.247	3.988	4.235	3.719	3.949	4.000	4.247	3.988	4.235	3.654	3.879	4.000	4.247	3.988	4.235	3.591	3.813
	10	0.7	0.035	0.019	0.035	0.019	0.035	0.019	0.035	0.019	0.035	0.019	0.035	0.019	0.035	0.019	0.035	0.019	0.035	0.019
		0.8	0.053	0.030	0.053	0.030	0.053	0.030	0.053	0.030	0.053	0.030	0.053	0.030	0.053	0.030	0.053	0.030	0.053	0.030
		0.9	0.339	0.356	0.339	0.356	0.339	0.356	0.339	0.356	0.339	0.356	0.338	0.355	0.339	0.356	0.339	0.356	0.338	0.355
		0.99	4.000	4.247	3.997	4.244	3.926	4.169	4.000	4.247	3.997	4.244	3.908	4.150	4.000	4.247	3.997	4.244	3.890	4.131

0.7	1	0.7	4.889	2.753	4.544	2.567	4.480	2.533	4.889	2.753	4.544	2.567	4.388	2.483	4.889	2.753	4.544	2.567	4.299	2.435
		0.8	7.282	4.247	6.501	3.803	6.363	3.724	7.282	4.247	6.501	3.803	6.165	3.612	7.282	4.247	6.501	3.803	5.977	3.505
		0.9	0.339	0.356	0.337	0.354	0.252	0.265	0.339	0.356	0.337	0.354	0.237	0.249	0.339	0.356	0.337	0.354	0.224	0.235
		0.99	4.000	4.247	3.714	3.968	0.608	0.641	4.000	4.247	3.714	3.968	0.498	0.524	4.000	4.247	3.714	3.968	0.426	0.446
	5	0.7	0.242	0.156	0.241	0.156	0.241	0.155	0.242	0.156	0.241	0.156	0.241	0.155	0.242	0.156	0.241	0.156	0.240	0.155
		0.8	0.362	0.233	0.360	0.232	0.360	0.231	0.362	0.233	0.360	0.232	0.359	0.231	0.362	0.233	0.360	0.232	0.359	0.231
		0.9	0.339	0.356	0.339	0.356	0.335	0.351	0.339	0.356	0.339	0.356	0.333	0.350	0.339	0.356	0.339	0.356	0.332	0.349
		0.99	4.000	4.247	3.988	4.235	3.394	3.603	4.000	4.247	3.988	4.235	3.267	3.468	4.000	4.247	3.988	4.235	3.148	3.341
	10	0.7	0.035	0.019	0.035	0.019	0.035	0.019	0.035	0.019	0.035	0.019	0.035	0.019	0.035	0.019	0.035	0.019	0.035	0.019
		0.8	0.053	0.030	0.053	0.030	0.053	0.030	0.053	0.030	0.053	0.030	0.053	0.030	0.053	0.030	0.053	0.030	0.053	0.030
		0.9	0.339	0.356	0.339	0.356	0.338	0.355	0.339	0.356	0.339	0.356	0.338	0.355	0.339	0.356	0.339	0.356	0.337	0.354
		0.99	4.000	4.247	3.997	4.244	3.832	4.068	4.000	4.247	3.997	4.244	3.791	4.026	4.000	4.247	3.997	4.244	3.752	3.984

0.9	1	0.7	4.889	2.753	4.454	2.519	4.375	2.476	4.889	2.753	4.454	2.519	4.261	2.415	4.889	2.753	4.454	2.519	4.153	2.356
		0.8	7.282	4.247	6.305	3.691	6.137	3.596	7.282	4.247	6.305	3.691	5.900	3.461	7.282	4.247	6.305	3.691	5.679	3.335
		0.9	0.339	0.356	0.337	0.354	0.235	0.247	0.339	0.356	0.337	0.354	0.219	0.229	0.339	0.356	0.337	0.354	0.204	0.214
		0.99	4.000	4.247	3.714	3.968	0.486	0.511	4.000	4.247	3.714	3.968	0.402	0.421	4.000	4.247	3.714	3.968	0.349	0.364
	5	0.7	0.242	0.156	0.241	0.155	0.240	0.155	0.242	0.156	0.241	0.155	0.240	0.155	0.242	0.156	0.241	0.155	0.240	0.155
		0.8	0.362	0.233	0.360	0.231	0.359	0.231	0.362	0.233	0.360	0.231	0.359	0.230	0.362	0.233	0.360	0.231	0.358	0.230
		0.9	0.339	0.356	0.339	0.356	0.333	0.350	0.339	0.356	0.339	0.356	0.332	0.349	0.339	0.356	0.339	0.356	0.330	0.347
		0.99	4.000	4.247	3.988	4.235	3.249	3.449	4.000	4.247	3.988	4.235	3.099	3.290	4.000	4.247	3.988	4.235	2.960	3.142
	10	0.7	0.035	0.019	0.035	0.019	0.035	0.019	0.035	0.019	0.035	0.019	0.035	0.019	0.035	0.019	0.035	0.019	0.035	0.019
		0.8	0.053	0.030	0.053	0.030	0.053	0.030	0.053	0.030	0.053	0.030	0.053	0.030	0.053	0.030	0.053	0.030	0.053	0.030
		0.9	0.339	0.356	0.339	0.356	0.338	0.355	0.339	0.356	0.339	0.356	0.337	0.354	0.339	0.356	0.339	0.356	0.330	0.347
		0.99	4.000	4.247	3.997	4.244	3.786	4.020	4.000	4.247	3.997	4.244	3.735	3.966	4.000	4.247	3.988	4.235	2.960	3.142

**Table 5:** Estimated MSEs of OLS, MRE, MRE, MRT, and RTMME at *n* = 50 and *p* = 3 when there is one outlier.

*k*	Sigma	Rho	*d* = 0.2						*d* = 0.5						*d* = 0.8					
			$\hat{\alpha }$	${\hat{\alpha }}_{M}$	$\hat{\alpha }\left(k\right)$	${\hat{\alpha }}_{M}\left(k\right)$	${\hat{\alpha }}_{\text{MRT}}$	${\hat{\alpha }}_{\text{RTMME}}$	$\hat{\alpha }$	${\hat{\alpha }}_{M}$	$\hat{\alpha }\left(k\right)$	${\hat{\alpha }}_{M}\left(k\right)$	${\hat{\alpha }}_{\text{MRT}}$	${\hat{\alpha }}_{\text{RTMME}}$	$\hat{\alpha }$	${\hat{\alpha }}_{M}$	$\hat{\alpha }\left(k\right)$	${\hat{\alpha }}_{M}\left(k\right)$	${\hat{\alpha }}_{\text{MRT}}$	${\hat{\alpha }}_{\text{RTMME}}$
0.3	1	0.7	5.240	2.879	5.081	2.795	5.051	2.779	5.240	2.879	5.081	2.795	5.005	2.755	5.240	2.879	5.081	2.795	4.961	2.731
		0.8	7.955	4.487	7.574	4.277	7.501	4.237	7.955	4.487	7.574	4.277	7.395	4.179	7.955	4.487	7.574	4.277	7.292	4.122
		0.9	37.725	0.362	37.365	0.360	32.709	0.318	37.725	0.362	37.365	0.360	31.629	0.309	37.725	0.362	37.365	0.360	30.609	0.300
		0.99	450.876	4.394	398.785	4.144	127.316	1.381	450.876	4.394	398.785	4.144	104.515	1.161	450.876	4.394	398.785	4.144	87.850	1.000
	5	0.7	0.332	0.185	0.332	0.185	0.332	0.185	0.332	0.185	0.332	0.185	0.332	0.185	0.355	0.201	0.355	0.201	0.354	0.201
		0.8	0.501	0.280	0.500	0.279	0.500	0.279	0.501	0.280	0.500	0.279	0.499	0.279	0.520	0.300	0.519	0.300	0.518	0.299
		0.9	7.783	0.362	7.781	0.362	7.739	0.360	7.783	0.362	7.781	0.362	7.728	0.359	7.783	0.362	7.781	0.362	7.717	0.359
		0.99	94.623	4.394	94.143	4.383	87.590	4.085	94.623	4.394	94.143	4.383	85.967	4.014	94.623	4.394	94.143	4.383	84.393	3.945
	10	0.7	0.039	0.020	0.039	0.020	0.039	0.020	0.039	0.020	0.039	0.020	0.039	0.020	0.039	0.020	0.039	0.020	0.039	0.020
		0.8	0.059	0.032	0.059	0.032	0.059	0.032	0.059	0.032	0.059	0.032	0.059	0.032	0.059	0.032	0.059	0.032	0.059	0.032
		0.9	4.049	0.362	4.049	0.362	4.043	0.361	4.049	0.362	4.049	0.362	4.042	0.361	4.049	0.362	4.049	0.362	4.041	0.361
		0.99	49.779	4.394	49.716	4.391	48.813	4.313	49.779	4.394	49.716	4.391	48.576	4.293	49.779	4.394	49.716	4.391	48.341	4.273

0.7	1	0.7	5.240	2.879	4.883	2.690	4.818	2.655	5.240	2.879	4.883	2.690	4.722	2.604	5.240	2.879	4.883	2.690	4.629	2.555
		0.8	7.955	4.487	7.115	4.024	6.966	3.942	7.955	4.487	7.115	4.024	6.753	3.824	7.955	4.487	7.115	4.024	6.551	3.713
		0.9	37.725	0.362	37.365	0.360	27.579	0.274	37.725	0.362	37.365	0.360	25.749	0.258	37.725	0.362	37.365	0.360	24.117	0.245
		0.99	450.876	4.394	398.785	4.144	55.219	0.684	450.876	4.394	398.785	4.144	42.745	0.563	450.876	4.394	398.785	4.144	34.339	0.484
	5	0.7	0.332	0.185	0.331	0.185	0.331	0.185	0.332	0.185	0.331	0.185	0.331	0.185	0.332	0.185	0.331	0.185	0.330	0.184
		0.8	0.501	0.280	0.499	0.278	0.498	0.278	0.501	0.280	0.499	0.278	0.497	0.278	0.501	0.280	0.499	0.278	0.497	0.277
		0.9	7.783	0.362	7.781	0.362	7.681	0.357	7.783	0.362	7.781	0.362	7.655	0.356	7.783	0.362	7.781	0.362	7.630	0.355
		0.99	94.623	4.394	94.143	4.383	79.478	3.728	94.623	4.394	94.143	4.383	76.315	3.589	94.623	4.394	94.143	4.383	73.357	3.458
	10	0.7	0.039	0.020	0.039	0.020	0.039	0.020	0.039	0.020	0.039	0.020	0.039	0.020	0.039	0.020	0.039	0.020	0.039	0.020
		0.8	0.059	0.032	0.059	0.032	0.059	0.032	0.059	0.032	0.059	0.032	0.059	0.032	0.059	0.032	0.059	0.032	0.059	0.032
		0.9	4.049	0.362	4.049	0.362	4.036	0.361	4.049	0.362	4.049	0.362	4.033	0.361	4.049	0.362	4.049	0.362	4.029	0.360
		0.99	49.779	4.394	49.716	4.391	47.572	4.209	49.779	4.394	49.716	4.391	47.046	4.165	49.779	4.394	49.716	4.391	46.529	4.122

0.9	1	0.7	5.240	2.879	4.790	2.640	4.708	2.597	5.240	2.879	4.790	2.640	4.591	2.534	5.240	2.879	4.790	2.640	4.479	2.475
		0.8	7.955	4.487	6.904	3.908	6.723	3.808	7.955	4.487	6.904	3.908	6.468	3.668	7.955	4.487	6.904	3.908	6.231	3.536
		0.9	37.725	0.362	37.365	0.360	25.505	0.256	37.725	0.362	37.365	0.360	23.472	0.239	37.725	0.362	37.365	0.360	21.702	0.225
		0.99	450.876	4.394	398.785	4.144	41.346	0.550	450.876	4.394	398.785	4.144	31.540	0.457	450.876	4.394	398.785	4.144	25.061	0.398
	5	0.7	0.332	0.185	0.331	0.185	0.331	0.185	0.332	0.185	0.331	0.185	0.330	0.184	0.332	0.185	0.331	0.185	0.330	0.184
		0.8	0.501	0.280	0.498	0.278	0.497	0.278	0.501	0.280	0.498	0.278	0.496	0.277	0.501	0.280	0.498	0.278	0.496	0.277
		0.9	7.783	0.362	7.781	0.362	7.652	0.356	7.783	0.362	7.781	0.362	7.619	0.355	7.783	0.362	7.781	0.362	7.587	0.353
		0.99	94.623	4.394	94.143	4.383	75.880	3.570	94.623	4.394	94.143	4.383	72.147	3.405	94.623	4.394	94.143	4.383	68.713	3.253
	10	0.7	0.039	0.020	0.039	0.020	0.039	0.020	0.039	0.020	0.039	0.020	0.039	0.020	0.039	0.020	0.039	0.020	0.039	0.020
		0.8	0.059	0.032	0.059	0.032	0.059	0.032	0.059	0.032	0.059	0.032	0.059	0.032	0.059	0.032	0.059	0.032	0.059	0.032
		0.9	4.049	0.362	4.049	0.362	4.032	0.360	4.049	0.362	4.049	0.362	4.028	0.360	7.783	0.362	7.781	0.362	7.587	0.353
		0.99	49.779	4.394	49.716	4.391	46.971	4.159	49.779	4.394	49.716	4.391	46.310	4.103	94.623	4.394	94.143	4.383	68.713	3.253

**Table 6:** Estimated MSEs of OLS, MRE, MRE, MRT, and RTMME at *n* = 50 and *p* = 3 when there are two outliers.

*k*	Sigma	Rho	*d* = 0.2						*d* = 0.5						*d* = 0.8					
			$\hat{\alpha }$	${\hat{\alpha }}_{M}$	$\hat{\alpha }\left(k\right)$	${\hat{\alpha }}_{M}\left(k\right)$	${\hat{\alpha }}_{\text{MRT}}$	${\hat{\alpha }}_{\text{RTMME}}$	$\hat{\alpha }$	${\hat{\alpha }}_{M}$	$\hat{\alpha }\left(k\right)$	${\hat{\alpha }}_{M}\left(k\right)$	${\hat{\alpha }}_{\text{MRT}}$	${\hat{\alpha }}_{\text{RTMME}}$	$\hat{\alpha }$	${\hat{\alpha }}_{M}$	$\hat{\alpha }\left(k\right)$	${\hat{\alpha }}_{M}\left(k\right)$	${\hat{\alpha }}_{\text{MRT}}$	${\hat{\alpha }}_{\text{RTMME}}$
0.3	1	0.7	5.896	3.408	5.700	3.298	5.662	3.277	5.896	3.408	5.700	3.298	5.606	3.246	5.896	3.408	5.700	3.298	5.551	3.215
		0.8	8.479	5.127	8.049	4.873	7.968	4.825	8.479	5.127	8.049	4.873	7.848	4.754	8.479	5.127	8.049	4.873	7.732	4.685
		0.9	14.066	0.399	13.883	0.396	13.240	0.350	14.066	0.399	13.883	0.396	13.047	0.340	14.066	0.399	13.883	0.396	12.860	0.330
		0.99	136.974	4.789	118.997	4.264	78.216	1.486	136.974	4.789	118.997	4.264	69.921	1.249	136.974	4.789	118.997	4.264	62.956	1.075
	5	0.7	0.355	0.201	0.355	0.201	0.354	0.201	0.355	0.201	0.355	0.201	0.354	0.201	0.355	0.201	0.355	0.201	0.354	0.201
		0.8	0.520	0.300	0.519	0.300	0.519	0.299	0.520	0.300	0.519	0.300	0.519	0.299	0.520	0.300	0.519	0.300	0.518	0.299
		0.9	3.085	0.399	3.083	0.399	3.077	0.397	3.085	0.399	3.083	0.399	3.076	0.396	3.085	0.399	3.083	0.399	3.074	0.396
		0.99	30.921	4.789	30.710	4.766	29.955	4.449	30.921	4.789	30.710	4.766	29.724	4.370	30.921	4.789	30.710	4.766	29.498	4.294
	10	0.7	0.051	0.025	0.051	0.025	0.051	0.025	0.051	0.025	0.051	0.025	0.051	0.025	0.051	0.025	0.051	0.025	0.051	0.025
		0.8	0.076	0.038	0.076	0.038	0.076	0.038	0.076	0.038	0.076	0.038	0.076	0.038	0.076	0.038	0.076	0.038	0.076	0.038
		0.9	1.712	0.399	1.712	0.399	1.711	0.398	1.712	0.399	1.712	0.399	1.711	0.398	1.712	0.399	1.712	0.399	1.710	0.398
		0.99	17.583	4.789	17.549	4.783	17.423	4.700	17.583	4.789	17.549	4.783	17.384	4.678	17.583	4.789	17.549	4.783	17.345	4.656

0.7	1	0.7	5.896	3.408	5.456	3.162	5.375	3.117	5.896	3.408	5.456	3.162	5.257	3.051	5.896	3.408	5.456	3.162	5.144	2.988
		0.8	8.479	5.127	7.533	4.567	7.366	4.468	8.479	5.127	7.533	4.567	7.127	4.327	8.479	5.127	7.533	4.567	6.902	4.193
		0.9	14.066	0.399	13.883	0.396	12.269	0.301	14.066	0.399	13.883	0.396	11.884	0.284	14.066	0.399	13.883	0.396	11.521	0.269
		0.99	136.974	4.789	118.997	4.264	46.238	0.735	136.974	4.789	118.997	4.264	38.368	0.606	136.974	4.789	118.997	4.264	32.440	0.521
	5	0.7	0.355	0.201	0.354	0.201	0.354	0.201	0.355	0.201	0.354	0.201	0.354	0.201	0.355	0.201	0.354	0.201	0.353	0.201
		0.8	0.520	0.300	0.518	0.299	0.517	0.298	0.520	0.300	0.518	0.299	0.517	0.298	0.520	0.300	0.518	0.299	0.516	0.298
		0.9	3.085	0.399	3.083	0.399	3.068	0.394	3.085	0.399	3.083	0.399	3.064	0.393	3.085	0.399	3.083	0.399	3.060	0.391
		0.99	30.921	4.789	30.710	4.766	28.770	4.056	30.921	4.789	30.710	4.766	28.284	3.902	30.921	4.789	30.710	4.766	27.816	3.758
	10	0.7	0.051	0.025	0.051	0.025	0.051	0.025	0.051	0.025	0.051	0.025	0.051	0.025	0.051	0.025	0.051	0.025	0.051	0.025
		0.8	0.076	0.038	0.076	0.038	0.076	0.038	0.076	0.038	0.076	0.038	0.076	0.038	0.076	0.038	0.076	0.038	0.075	0.038
		0.9	1.712	0.399	1.712	0.399	1.709	0.398	1.712	0.399	1.712	0.399	1.709	0.397	1.712	0.399	1.712	0.399	1.708	0.397
		0.99	17.583	4.789	17.549	4.783	17.216	4.585	17.583	4.789	17.549	4.783	17.128	4.537	17.583	4.789	17.549	4.783	17.040	4.489

0.9	1	0.7	5.896	3.408	5.341	3.098	5.241	3.042	5.896	3.408	5.341	3.098	5.097	2.962	5.896	3.408	5.341	3.098	4.960	2.885
		0.8	8.479	5.127	7.296	4.427	7.094	4.307	8.479	5.127	7.296	4.427	6.809	4.139	8.479	5.127	7.296	4.427	6.544	3.981
		0.9	14.066	0.399	13.883	0.396	11.831	0.282	14.066	0.399	13.883	0.396	11.371	0.263	14.066	0.399	13.883	0.396	10.943	0.247
		0.99	136.974	4.789	118.997	4.264	37.419	0.592	136.974	4.789	118.997	4.264	30.337	0.493	136.974	4.789	118.997	4.264	25.185	0.429
	5	0.7	0.355	0.201	0.354	0.201	0.354	0.201	0.355	0.201	0.354	0.201	0.353	0.200	0.355	0.201	0.354	0.201	0.353	0.200
		0.8	0.520	0.300	0.517	0.298	0.517	0.298	0.520	0.300	0.517	0.298	0.516	0.297	0.520	0.300	0.517	0.298	0.515	0.297
		0.9	3.085	0.399	3.083	0.399	3.063	0.393	3.085	0.399	3.083	0.399	3.058	0.391	3.085	0.399	3.083	0.399	3.053	0.389
		0.99	30.921	4.789	30.710	4.766	28.216	3.881	30.921	4.789	30.710	4.766	27.620	3.699	30.921	4.789	30.710	4.766	27.051	3.532
	10	0.7	0.051	0.025	0.051	0.025	0.051	0.025	0.051	0.025	0.051	0.025	0.051	0.025	0.051	0.025	0.051	0.025	0.051	0.025
		0.8	0.076	0.038	0.076	0.038	0.075	0.038	0.076	0.038	0.076	0.038	0.075	0.038	0.076	0.038	0.076	0.038	0.075	0.038
		0.9	1.712	0.399	1.712	0.399	1.709	0.397	1.712	0.399	1.712	0.399	1.708	0.397	3.085	0.399	3.083	0.399	3.053	0.389
		0.99	17.583	4.789	17.549	4.783	17.115	4.530	17.583	4.789	17.549	4.783	17.003	4.469	30.921	4.789	30.710	4.766	27.051	3.532

**Table 7:** Estimated MSEs of OLS, MRE, MRE, MRT, and RTMME at *n* = 100 and *p* = 3 when there is no outlier.

*k*	Sigma	Rho	*d* = 0.2						*d* = 0.5						*d* = 0.8					
			$\hat{\alpha }$	${\hat{\alpha }}_{M}$	$\hat{\alpha }\left(k\right)$	${\hat{\alpha }}_{M}\left(k\right)$	${\hat{\alpha }}_{\text{MRT}}$	${\hat{\alpha }}_{\text{RTMME}}$	$\hat{\alpha }$	${\hat{\alpha }}_{M}$	$\hat{\alpha }\left(k\right)$	${\hat{\alpha }}_{M}\left(k\right)$	${\hat{\alpha }}_{\text{MRT}}$	${\hat{\alpha }}_{\text{RTMME}}$	$\hat{\alpha }$	${\hat{\alpha }}_{M}$	$\hat{\alpha }\left(k\right)$	${\hat{\alpha }}_{M}\left(k\right)$	${\hat{\alpha }}_{\text{MRT}}$	${\hat{\alpha }}_{\text{RTMME}}$
0.3	1	0.7	3.350	2.231	3.289	2.188	3.277	2.180	3.350	2.231	3.289	2.188	3.259	2.168	3.350	2.231	3.289	2.188	3.241	2.155
		0.8	4.756	3.200	4.621	3.106	4.594	3.088	4.756	3.200	4.621	3.106	4.555	3.061	4.756	3.200	4.621	3.106	4.517	3.035
		0.9	0.189	0.200	0.188	0.199	0.176	0.186	0.189	0.200	0.188	0.199	0.173	0.183	0.189	0.200	0.188	0.199	0.170	0.180
		0.99	2.079	2.210	2.005	2.137	1.034	1.100	2.079	2.210	2.005	2.137	0.908	0.966	2.079	2.210	2.005	2.137	0.807	0.858
	5	0.7	0.094	0.046	0.094	0.046	0.094	0.046	0.094	0.046	0.094	0.046	0.094	0.046	0.094	0.046	0.094	0.046	0.094	0.046
		0.8	0.134	0.065	0.133	0.065	0.133	0.065	0.134	0.065	0.133	0.065	0.133	0.065	0.134	0.065	0.133	0.065	0.133	0.065
		0.9	0.189	0.200	0.189	0.200	0.188	0.199	0.189	0.200	0.189	0.200	0.188	0.199	0.189	0.200	0.189	0.200	0.188	0.199
		0.99	2.079	2.210	2.076	2.207	2.006	2.132	2.079	2.210	2.076	2.207	1.989	2.113	2.079	2.210	2.076	2.207	1.972	2.095
	10	0.7	0.022	0.012	0.022	0.012	0.022	0.012	0.022	0.012	0.022	0.012	0.022	0.012	0.022	0.012	0.022	0.012	0.022	0.012
		0.8	0.032	0.018	0.032	0.018	0.032	0.018	0.032	0.018	0.032	0.018	0.032	0.018	0.032	0.018	0.032	0.018	0.032	0.018
		0.9	0.189	0.200	0.189	0.200	0.189	0.199	0.189	0.200	0.189	0.200	0.189	0.199	0.189	0.200	0.189	0.200	0.189	0.199
		0.99	2.079	2.210	2.079	2.209	2.061	2.190	2.079	2.210	2.079	2.209	2.056	2.185	2.079	2.210	2.079	2.209	2.051	2.180

0.7	1	0.7	3.350	2.231	3.210	2.134	3.183	2.115	3.350	2.231	3.210	2.134	3.143	2.088	3.350	2.231	3.289	2.188	3.241	2.155
		0.8	4.756	3.200	4.450	2.988	4.392	2.948	4.756	3.200	4.450	2.988	4.308	2.891	4.756	3.200	4.621	3.106	4.517	3.035
		0.9	0.189	0.200	0.188	0.199	0.161	0.170	0.189	0.200	0.188	0.199	0.156	0.164	0.189	0.200	0.188	0.199	0.150	0.159
		0.99	2.079	2.210	2.005	2.137	0.583	0.620	2.079	2.210	2.005	2.137	0.487	0.517	2.079	2.210	2.005	2.137	0.419	0.445
	5	0.7	0.094	0.046	0.094	0.046	0.094	0.046	0.094	0.046	0.094	0.046	0.094	0.046	0.094	0.046	0.094	0.046	0.094	0.046
		0.8	0.134	0.065	0.133	0.064	0.133	0.064	0.134	0.065	0.133	0.064	0.133	0.064	0.134	0.065	0.133	0.065	0.133	0.065
		0.9	0.189	0.200	0.189	0.200	0.188	0.198	0.189	0.200	0.189	0.200	0.187	0.198	0.189	0.200	0.189	0.200	0.187	0.198
		0.99	2.079	2.210	2.076	2.207	1.916	2.036	2.079	2.210	2.076	2.207	1.878	1.996	2.079	2.210	2.076	2.207	1.842	1.957
	10	0.7	0.022	0.012	0.022	0.012	0.022	0.012	0.022	0.012	0.022	0.012	0.022	0.012	0.022	0.012	0.022	0.012	0.022	0.012
		0.8	0.032	0.018	0.032	0.018	0.032	0.018	0.032	0.018	0.032	0.018	0.032	0.018	0.032	0.018	0.032	0.018	0.032	0.018
		0.9	0.189	0.200	0.189	0.200	0.189	0.199	0.189	0.200	0.189	0.200	0.188	0.199	0.189	0.200	0.189	0.200	0.188	0.199
		0.99	2.079	2.210	2.079	2.209	2.036	2.164	2.079	2.210	2.079	2.209	2.026	2.153	2.079	2.210	2.079	2.209	2.015	2.142

0.9	1	0.7	0.175	0.185	0.165	0.175	0.163	0.173	0.175	0.185	0.165	0.175	0.161	0.170	0.175	0.185	0.165	0.175	0.159	0.168
		0.8	0.276	0.293	0.251	0.266	0.247	0.262	0.276	0.293	0.251	0.266	0.241	0.255	0.276	0.293	0.251	0.266	0.236	0.250
		0.9	0.189	0.200	0.188	0.199	0.155	0.164	0.189	0.200	0.188	0.199	0.148	0.156	0.189	0.200	0.188	0.199	0.142	0.150
		0.99	2.079	2.210	2.005	2.137	0.476	0.505	2.079	2.210	2.005	2.137	0.396	0.420	2.079	2.210	2.005	2.137	0.342	0.361
	5	0.7	0.150	0.158	0.149	0.158	0.149	0.158	0.150	0.158	0.149	0.158	0.149	0.158	0.150	0.158	0.149	0.158	0.149	0.157
		0.8	0.229	0.242	0.228	0.241	0.228	0.241	0.229	0.242	0.228	0.241	0.228	0.240	0.229	0.242	0.228	0.241	0.227	0.240
		0.9	0.189	0.200	0.189	0.200	0.187	0.198	0.189	0.200	0.189	0.200	0.187	0.197	0.189	0.200	0.189	0.200	0.186	0.197
		0.99	2.079	2.210	2.076	2.207	1.873	1.990	2.079	2.210	2.076	2.207	1.827	1.941	2.079	2.210	2.076	2.207	1.782	1.894
	10	0.7	0.156	0.164	0.156	0.164	0.155	0.163	0.156	0.164	0.156	0.164	0.155	0.163	0.156	0.164	0.156	0.164	0.155	0.163
		0.8	0.229	0.243	0.229	0.243	0.229	0.243	0.229	0.243	0.229	0.243	0.229	0.243	0.229	0.243	0.229	0.243	0.229	0.243
		0.9	0.189	0.200	0.189	0.200	0.188	0.199	0.189	0.200	0.189	0.200	0.188	0.199	0.189	0.200	0.189	0.200	0.188	0.199
		0.99	2.079	2.210	2.079	2.209	2.024	2.151	2.079	2.210	2.079	2.209	2.011	2.137	2.079	2.210	2.079	2.209	1.998	2.123

**Table 8:** Estimated MSEs of OLS, MRE, MRE, MRT, and RTMME at *n* = 100 and *p* = 3 when there is one outlier.

*k*	Sigma	Rho	*d* = 0.2						*d* = 0.5						*d* = 0.8					
			$\hat{\alpha }$	${\hat{\alpha }}_{M}$	$\hat{\alpha }\left(k\right)$	${\hat{\alpha }}_{M}\left(k\right)$	${\hat{\alpha }}_{\text{MRT}}$	${\hat{\alpha }}_{\text{RTMME}}$	$\hat{\alpha }$	${\hat{\alpha }}_{M}$	$\hat{\alpha }\left(k\right)$	${\hat{\alpha }}_{M}\left(k\right)$	${\hat{\alpha }}_{\text{MRT}}$	${\hat{\alpha }}_{\text{RTMME}}$	$\hat{\alpha }$	${\hat{\alpha }}_{M}$	$\hat{\alpha }\left(k\right)$	${\hat{\alpha }}_{M}\left(k\right)$	${\hat{\alpha }}_{\text{MRT}}$	${\hat{\alpha }}_{\text{RTMME}}$
0.3	1	0.7	3.404	2.252	3.343	2.210	3.331	2.201	3.404	2.252	3.343	2.210	3.313	2.189	3.404	2.252	3.343	2.210	3.296	2.177
		0.8	4.821	3.230	4.686	3.136	4.660	3.118	4.821	3.230	4.686	3.136	4.621	3.091	4.821	3.230	4.686	3.136	4.583	3.064
		0.9	5.532	0.201	5.516	0.200	5.287	0.187	5.532	0.201	5.516	0.200	5.229	0.184	5.532	0.201	5.516	0.200	5.173	0.181
		0.99	69.936	2.224	66.795	2.161	39.834	1.114	69.936	2.224	66.795	2.161	35.815	0.980	69.936	2.224	66.795	2.161	32.482	0.873
	5	0.7	0.100	0.047	0.100	0.047	0.100	0.047	0.100	0.047	0.100	0.047	0.100	0.047	0.100	0.047	0.100	0.047	0.100	0.047
		0.8	0.144	0.068	0.144	0.067	0.144	0.067	0.144	0.068	0.144	0.067	0.144	0.067	0.144	0.068	0.144	0.067	0.144	0.067
		0.9	1.261	0.201	1.261	0.201	1.259	0.200	1.261	0.201	1.261	0.201	1.258	0.200	1.261	0.201	1.261	0.201	1.258	0.200
		0.99	15.725	2.223	15.695	2.221	15.269	2.146	15.725	2.223	15.695	2.221	15.158	2.127	15.725	2.223	15.695	2.221	15.050	2.109
	10	0.7	0.030	0.014	0.030	0.014	0.030	0.014	0.030	0.014	0.030	0.014	0.030	0.014	0.030	0.014	0.030	0.014	0.030	0.014
		0.8	0.045	0.021	0.045	0.021	0.045	0.021	0.045	0.021	0.045	0.021	0.045	0.021	0.045	0.021	0.045	0.021	0.045	0.021
		0.9	0.726	0.201	0.726	0.201	0.726	0.201	0.726	0.201	0.726	0.201	0.726	0.200	0.726	0.201	0.726	0.201	0.726	0.200
		0.99	8.930	2.223	8.926	2.223	8.862	2.204	8.930	2.223	8.926	2.223	8.846	2.199	8.930	2.223	8.926	2.223	8.829	2.194

0.7	1	0.7	3.404	2.252	3.265	2.155	3.238	2.137	3.404	2.252	3.265	2.155	3.199	2.110	3.404	2.252	3.343	2.210	3.296	2.177
		0.8	4.821	3.230	4.516	3.018	4.459	2.978	4.821	3.230	4.516	3.018	4.376	2.920	4.821	3.230	4.686	3.136	4.583	3.064
		0.9	5.532	0.201	5.516	0.200	4.994	0.172	5.532	0.201	5.516	0.200	4.877	0.167	5.532	0.201	5.516	0.200	4.765	0.161
		0.99	69.936	2.224	66.795	2.161	24.567	0.637	69.936	2.224	66.795	2.161	20.838	0.535	69.936	2.224	66.795	2.161	18.001	0.462
	5	0.7	0.100	0.047	0.100	0.047	0.100	0.047	0.100	0.047	0.100	0.047	0.100	0.047	0.100	0.047	0.100	0.047	0.100	0.047
		0.8	0.144	0.068	0.143	0.067	0.143	0.067	0.144	0.068	0.143	0.067	0.143	0.067	0.144	0.068	0.144	0.067	0.144	0.067
		0.9	1.261	0.201	1.261	0.201	1.256	0.199	1.261	0.201	1.261	0.201	1.255	0.199	1.261	0.201	1.261	0.201	1.253	0.199
		0.99	15.725	2.223	15.695	2.221	14.698	2.049	15.725	2.223	15.695	2.221	14.461	2.009	15.725	2.223	15.695	2.221	14.232	1.971
	10	0.7	0.030	0.014	0.030	0.014	0.030	0.014	0.030	0.014	0.030	0.014	0.030	0.014	0.030	0.014	0.030	0.014	0.030	0.014
		0.8	0.045	0.021	0.045	0.021	0.045	0.021	0.045	0.021	0.045	0.021	0.045	0.021	0.045	0.021	0.045	0.021	0.045	0.021
		0.9	0.726	0.201	0.726	0.201	0.726	0.200	0.726	0.201	0.726	0.201	0.725	0.200	0.726	0.201	0.726	0.201	0.725	0.200
		0.99	8.930	2.223	8.926	2.223	8.774	2.178	8.930	2.223	8.926	2.223	8.735	2.166	8.930	2.223	8.926	2.223	8.697	2.155

0.9	1	0.7	0.414	0.187	0.395	0.177	0.391	0.175	0.414	0.187	0.395	0.177	0.387	0.172	0.414	0.187	0.395	0.177	0.382	0.170
		0.8	0.606	0.295	0.564	0.268	0.556	0.264	0.606	0.295	0.564	0.268	0.546	0.258	0.606	0.295	0.564	0.268	0.536	0.252
		0.9	5.532	0.201	5.516	0.200	4.860	0.166	5.532	0.201	5.516	0.200	4.719	0.159	5.532	0.201	5.516	0.200	4.586	0.153
		0.99	69.936	2.224	66.795	2.161	20.386	0.523	5.532	2.224	66.795	2.161	16.984	0.438	69.936	2.224	66.795	2.161	14.463	0.380
	5	0.7	0.211	0.160	0.211	0.160	0.211	0.160	5.532	0.160	0.211	0.160	0.210	0.160	0.211	0.160	0.211	0.160	0.210	0.160
		0.8	0.316	0.246	0.315	0.245	0.315	0.245	5.532	0.246	0.315	0.245	0.314	0.244	0.316	0.246	0.315	0.245	0.314	0.244
		0.9	1.261	0.201	1.261	0.201	1.255	0.199	5.532	0.201	1.261	0.201	1.253	0.199	1.261	0.201	1.261	0.201	1.251	0.198
		0.99	15.725	2.223	15.695	2.221	14.428	2.004	5.532	2.223	15.695	2.221	14.136	1.955	15.725	2.223	15.695	2.221	13.854	1.907
	10	0.7	0.199	0.168	0.199	0.168	0.199	0.168	5.532	0.168	0.199	0.168	0.199	0.168	0.199	0.168	0.199	0.168	0.199	0.168
		0.8	0.295	0.249	0.295	0.248	0.295	0.248	5.532	0.249	0.295	0.248	0.294	0.248	0.295	0.249	0.295	0.248	0.294	0.248
		0.9	0.726	0.201	0.726	0.201	0.725	0.200	5.532	0.201	0.726	0.201	0.725	0.200	0.726	0.201	0.726	0.201	0.725	0.200
		0.99	8.930	2.223	8.926	2.223	8.730	2.165	5.532	2.223	8.926	2.223	8.681	2.151	8.930	2.223	8.926	2.223	8.633	2.136

**Table 9:** Estimated MSEs of OLS, MRE, MRE, MRT, and RTMME at *n* = 100 and *p* = 3 when there are two outliers.

*k*	Sigma	Rho	*d* = 0.2						*d* = 0.5						*d* = 0.8					
			$\hat{\alpha }$	${\hat{\alpha }}_{M}$	$\hat{\alpha }\left(k\right)$	${\hat{\alpha }}_{M}\left(k\right)$	${\hat{\alpha }}_{\text{MRT}}$	${\hat{\alpha }}_{\text{RTMME}}$	$\hat{\alpha }$	${\hat{\alpha }}_{M}$	$\hat{\alpha }\left(k\right)$	${\hat{\alpha }}_{M}\left(k\right)$	${\hat{\alpha }}_{\text{MRT}}$	${\hat{\alpha }}_{\text{RTMME}}$	$\hat{\alpha }$	${\hat{\alpha }}_{M}$	$\hat{\alpha }\left(k\right)$	${\hat{\alpha }}_{M}\left(k\right)$	${\hat{\alpha }}_{\text{MRT}}$	${\hat{\alpha }}_{\text{RTMME}}$
0.3	1	0.7	3.529	2.360	3.463	2.316	3.450	2.307	3.529	2.360	3.463	2.316	3.431	2.294	3.529	2.360	3.463	2.316	3.412	2.281
		0.8	5.046	3.388	4.901	3.289	4.873	3.269	5.046	3.388	4.901	3.289	4.831	3.241	5.046	3.388	4.901	3.289	4.790	3.212
		0.9	2.737	0.207	2.706	0.206	2.596	0.194	2.737	0.207	2.706	0.206	2.563	0.191	2.737	0.207	2.706	0.206	2.531	0.188
		0.99	33.739	2.273	28.776	2.151	18.246	1.161	33.739	2.273	28.776	2.151	16.274	1.026	33.739	2.273	28.776	2.151	14.661	0.918
	5	0.7	0.099	0.049	0.099	0.049	0.099	0.049	0.099	0.049	0.099	0.049	0.099	0.049	0.099	0.049	0.099	0.049	0.099	0.049
		0.8	0.140	0.070	0.140	0.070	0.140	0.070	0.140	0.070	0.140	0.070	0.140	0.070	0.140	0.070	0.140	0.070	0.140	0.070
		0.9	0.702	0.207	0.702	0.207	0.701	0.206	0.702	0.207	0.702	0.207	0.700	0.206	0.702	0.207	0.702	0.207	0.700	0.206
		0.99	8.471	2.273	8.413	2.267	8.201	2.195	8.471	2.273	8.413	2.267	8.135	2.176	8.471	2.273	8.413	2.267	8.071	2.158
	10	0.7	0.024	0.015	0.024	0.015	0.024	0.015	0.024	0.015	0.024	0.015	0.024	0.015	0.024	0.015	0.024	0.015	0.024	0.015
		0.8	0.034	0.021	0.034	0.021	0.034	0.021	0.034	0.021	0.034	0.021	0.034	0.021	0.034	0.021	0.034	0.021	0.034	0.021
		0.9	0.447	0.207	0.447	0.207	0.447	0.207	0.447	0.207	0.447	0.207	0.447	0.207	0.447	0.207	0.447	0.207	0.447	0.207
		0.99	5.298	2.273	5.289	2.271	5.254	2.253	5.298	2.273	5.289	2.271	5.243	2.248	5.298	2.273	5.289	2.271	5.232	2.243

0.7	1	0.7	3.529	2.360	3.378	2.258	3.349	2.238	3.529	2.360	3.378	2.258	3.307	2.209	3.529	2.360	3.463	2.316	3.412	2.281
		0.8	5.046	3.388	4.718	3.163	4.656	3.121	5.046	3.388	4.718	3.163	4.566	3.059	5.046	3.388	4.901	3.289	4.790	3.212
		0.9	2.737	0.207	2.706	0.206	2.431	0.179	2.737	0.207	2.706	0.206	2.365	0.173	2.737	0.207	2.706	0.206	2.304	0.168
		0.99	33.739	2.273	28.776	2.151	10.921	0.678	33.739	2.273	28.776	2.151	9.207	0.574	33.739	2.273	28.776	2.151	7.924	0.499
	5	0.7	0.099	0.049	0.099	0.049	0.099	0.049	0.099	0.049	0.099	0.049	0.099	0.049	0.099	0.049	0.099	0.049	0.099	0.049
		0.8	0.140	0.070	0.140	0.070	0.140	0.070	0.140	0.070	0.140	0.070	0.140	0.070	0.140	0.070	0.140	0.070	0.140	0.070
		0.9	0.702	0.207	0.702	0.207	0.699	0.206	0.702	0.207	0.702	0.207	0.698	0.205	0.702	0.207	0.702	0.207	0.697	0.205
		0.99	8.471	2.273	8.413	2.267	7.864	2.099	8.471	2.273	8.413	2.267	7.724	2.059	8.471	2.273	8.413	2.267	7.589	2.020
	10	0.7	0.024	0.015	0.024	0.015	0.024	0.015	0.024	0.015	0.024	0.015	0.024	0.015	0.024	0.015	0.024	0.015	0.024	0.015
		0.8	0.034	0.021	0.034	0.021	0.034	0.021	0.034	0.021	0.034	0.021	0.034	0.021	0.034	0.021	0.034	0.021	0.034	0.021
		0.9	0.447	0.207	0.447	0.207	0.446	0.207	0.447	0.207	0.447	0.207	0.446	0.207	0.447	0.207	0.447	0.207	0.446	0.206
		0.99	5.298	2.273	5.289	2.271	5.196	2.227	5.298	2.273	5.289	2.271	5.171	2.216	5.298	2.273	5.289	2.271	5.146	2.204

0.9	1	0.7	0.835	0.192	0.811	0.182	0.807	0.180	0.835	0.192	0.811	0.182	0.800	0.178	0.835	0.192	0.811	0.182	0.794	0.176
		0.8	1.220	0.302	1.163	0.276	1.153	0.271	1.220	0.302	1.163	0.276	1.138	0.265	1.220	0.302	1.163	0.276	1.123	0.260
		0.9	2.737	0.207	2.706	0.206	2.356	0.172	2.737	0.207	2.706	0.206	2.278	0.166	2.737	0.207	2.706	0.206	2.205	0.159
		0.99	33.739	2.273	28.776	2.151	9.001	0.561	33.739	2.273	28.776	2.151	7.469	0.473	33.739	2.273	28.776	2.151	6.351	0.413
	5	0.7	0.264	0.163	0.264	0.162	0.263	0.162	0.264	0.163	0.264	0.162	0.263	0.162	0.264	0.163	0.264	0.162	0.263	0.162
		0.8	0.401	0.251	0.399	0.249	0.398	0.249	0.401	0.251	0.399	0.249	0.398	0.249	0.401	0.251	0.399	0.249	0.397	0.249
		0.9	0.702	0.207	0.702	0.207	0.698	0.205	0.702	0.207	0.702	0.207	0.697	0.205	0.702	0.207	0.702	0.207	0.696	0.205
		0.99	8.471	2.273	8.413	2.267	7.704	2.053	8.471	2.273	8.413	2.267	7.532	2.004	8.471	2.273	8.413	2.267	7.367	1.957
	10	0.7	0.311	0.178	0.311	0.178	0.311	0.178	0.311	0.178	0.311	0.178	0.311	0.178	0.311	0.178	0.311	0.178	0.311	0.178
		0.8	0.463	0.265	0.462	0.265	0.462	0.265	0.463	0.265	0.462	0.265	0.462	0.265	0.463	0.265	0.462	0.265	0.462	0.265
		0.9	0.447	0.207	0.447	0.207	0.446	0.207	0.447	0.207	0.447	0.207	0.446	0.206	0.447	0.207	0.447	0.207	0.446	0.206
		0.99	5.298	2.273	5.289	2.271	5.168	2.214	5.298	2.273	5.289	2.271	5.136	2.200	5.298	2.273	5.289	2.271	5.105	2.186

**Table 10:** Estimated MSEs of OLS, MRE, MRE, MRT, and RTMME at *n* = 20 and *p* = 7 when there is no outlier.

*k*	Sigma	Rho	*d* = 0.2						*d* = 0.5						*d* = 0.8					
			$\hat{\alpha }$	${\hat{\alpha }}_{M}$	$\hat{\alpha }\left(k\right)$	${\hat{\alpha }}_{M}\left(k\right)$	${\hat{\alpha }}_{\text{MRT}}$	${\hat{\alpha }}_{\text{RTMME}}$	$\hat{\alpha }$	${\hat{\alpha }}_{M}$	$\hat{\alpha }\left(k\right)$	${\hat{\alpha }}_{M}\left(k\right)$	${\hat{\alpha }}_{\text{MRT}}$	${\hat{\alpha }}_{\text{RTMME}}$	$\hat{\alpha }$	${\hat{\alpha }}_{M}$	$\hat{\alpha }\left(k\right)$	${\hat{\alpha }}_{M}\left(k\right)$	${\hat{\alpha }}_{\text{MRT}}$	${\hat{\alpha }}_{\text{RTMME}}$
0.3	1	0.7	1.430	1.543	1.140	1.228	1.098	1.182	1.430	1.543	1.140	1.228	1.041	1.120	1.430	1.543	1.140	1.228	0.991	1.066
		0.8	2.160	2.320	1.529	1.641	1.449	1.555	2.160	2.320	1.529	1.641	1.345	1.443	2.160	2.320	1.529	1.641	1.258	1.349
		0.9	4.469	4.885	2.335	2.534	2.145	2.326	4.469	4.885	2.335	2.534	1.918	2.076	4.469	4.885	2.335	2.534	1.739	1.880
		0.99	56.157	59.856	4.310	4.575	3.580	3.798	56.157	59.856	4.310	4.575	2.829	2.998	56.157	59.856	4.310	4.575	2.323	2.458
	5	0.7	1.389	1.492	1.374	1.476	1.372	1.473	1.389	1.492	1.374	1.476	1.367	1.468	1.389	1.492	1.374	1.476	1.363	1.464
		0.8	2.093	2.267	2.059	2.229	2.052	2.222	2.093	2.267	2.059	2.229	2.042	2.211	2.093	2.267	2.059	2.229	2.032	2.200
		0.9	4.316	4.637	4.157	4.466	4.127	4.434	4.316	4.637	4.157	4.466	4.082	4.386	4.316	4.637	4.157	4.466	4.038	4.339
		0.99	48.687	53.009	31.919	34.605	29.918	32.414	48.687	53.009	31.919	34.605	27.380	29.636	48.687	53.009	31.919	34.605	25.271	27.331
	10	0.7	1.034	1.107	1.032	1.105	1.032	1.105	1.034	1.107	1.032	1.105	1.031	1.104	1.034	1.107	1.032	1.105	1.031	1.104
		0.8	1.561	1.679	1.558	1.675	1.557	1.674	1.561	1.679	1.558	1.675	1.556	1.672	1.561	1.679	1.558	1.675	1.555	1.671
		0.9	3.151	3.404	3.134	3.385	3.130	3.381	3.151	3.404	3.134	3.385	3.125	3.375	3.151	3.404	3.134	3.385	3.120	3.370
		0.99	34.931	37.511	32.402	34.797	31.942	34.304	34.931	37.511	32.402	34.797	31.279	33.592	34.931	37.511	32.402	34.797	30.644	32.911

0.7	1	0.7	1.430	1.543	0.915	0.983	0.860	0.923	1.430	1.543	0.915	0.983	0.792	0.849	1.430	1.543	0.915	0.983	0.738	0.790
		0.8	2.160	2.320	1.132	1.213	1.044	1.118	2.160	2.320	1.132	1.213	0.940	1.005	2.160	2.320	1.132	1.213	0.859	0.917
		0.9	4.469	4.885	1.499	1.617	1.342	1.445	4.469	4.885	1.499	1.617	1.166	1.252	4.469	4.885	1.499	1.617	1.036	1.110
		0.99	56.157	59.856	1.751	1.848	1.439	1.516	56.157	59.856	1.751	1.848	1.145	1.201	56.157	59.856	1.751	1.848	0.963	1.006
	5	0.7	1.389	1.492	1.356	1.456	1.349	1.449	1.389	1.492	1.356	1.456	1.340	1.439	1.389	1.492	1.356	1.456	1.330	1.429
		0.8	2.093	2.267	2.014	2.181	1.999	2.164	2.093	2.267	2.014	2.181	1.977	2.140	2.093	2.267	2.014	2.181	1.955	2.116
		0.9	4.316	4.637	3.963	4.258	3.899	4.190	4.316	4.637	3.963	4.258	3.808	4.092	4.316	4.637	3.963	4.258	3.721	3.998
		0.99	48.687	53.009	22.287	24.073	20.245	21.847	48.687	53.009	22.287	24.073	17.850	19.242	48.687	53.009	22.287	24.073	16.002	17.234
	10	0.7	1.034	1.107	1.030	1.103	1.029	1.102	1.034	1.107	1.030	1.103	1.028	1.101	1.034	1.107	1.030	1.103	1.027	1.100
		0.8	1.561	1.679	1.553	1.669	1.551	1.667	1.561	1.679	1.553	1.669	1.548	1.664	1.561	1.679	1.553	1.669	1.546	1.662
		0.9	3.151	3.404	3.111	3.360	3.103	3.351	3.151	3.404	3.111	3.360	3.091	3.338	3.151	3.404	3.111	3.360	3.079	3.325
		0.99	34.931	37.511	29.583	31.771	28.717	30.842	34.931	37.511	29.583	31.771	27.518	29.555	34.931	37.511	29.583	31.771	26.424	28.380

0.9	1	0.7	1.430	1.543	0.839	0.901	0.784	0.840	1.430	1.543	0.839	0.901	0.717	0.767	1.430	1.543	0.839	0.901	0.665	0.710
		0.8	2.160	2.320	1.011	1.082	0.927	0.991	2.160	2.320	1.011	1.082	0.829	0.885	2.160	2.320	1.011	1.082	0.755	0.804
		0.9	4.469	4.885	1.286	1.384	1.145	1.229	4.469	4.885	1.286	1.384	0.990	1.059	4.469	4.885	1.286	1.384	0.877	0.936
		0.99	56.157	59.856	1.339	1.409	1.113	1.167	56.157	59.856	1.339	1.409	0.906	0.945	56.157	59.856	1.339	1.409	0.782	0.811
	5	0.7	1.389	1.492	1.347	1.446	1.338	1.437	1.389	1.492	1.347	1.446	1.326	1.424	1.389	1.492	1.347	1.446	1.315	1.412
		0.8	2.093	2.267	1.993	2.157	1.974	2.136	2.093	2.267	1.993	2.157	1.946	2.106	2.093	2.267	1.993	2.157	1.919	2.077
		0.9	4.316	4.637	3.873	4.161	3.795	4.078	4.316	4.637	3.873	4.161	3.684	3.959	4.316	4.637	3.873	4.161	3.581	3.848
		0.99	48.687	53.009	19.490	21.026	17.558	18.924	48.687	53.009	19.490	21.026	15.332	16.507	48.687	53.009	19.490	21.026	13.640	14.673
	10	0.7	1.034	1.107	1.029	1.102	1.028	1.101	1.034	1.107	1.029	1.102	1.027	1.099	1.034	1.107	1.029	1.102	1.025	1.098
		0.8	1.561	1.679	1.550	1.666	1.548	1.664	1.561	1.679	1.550	1.666	1.545	1.660	1.561	1.679	1.550	1.666	1.541	1.657
		0.9	3.151	3.404	3.099	3.347	3.089	3.336	3.151	3.404	3.099	3.347	3.074	3.320	3.151	3.404	3.099	3.347	3.059	3.304
		0.99	34.931	37.511	28.363	30.462	27.356	29.380	34.931	37.511	28.363	30.462	25.983	27.907	34.931	37.511	28.363	30.462	24.754	26.587

**Table 11:** Estimated MSEs of OLS, MRE, MRE, MRT, and RTMME at *n* = 20 and *p* = 7 when there is one outlier.

*k*	Sigma	Rho	*d* = 0.2						*d* = 0.5						*d* = 0.8					
			$\hat{\alpha }$	${\hat{\alpha }}_{M}$	$\hat{\alpha }\left(k\right)$	${\hat{\alpha }}_{M}\left(k\right)$	${\hat{\alpha }}_{\text{MRT}}$	${\hat{\alpha }}_{\text{RTMME}}$	$\hat{\alpha }$	${\hat{\alpha }}_{M}$	$\hat{\alpha }\left(k\right)$	${\hat{\alpha }}_{M}\left(k\right)$	${\hat{\alpha }}_{\text{MRT}}$	${\hat{\alpha }}_{\text{RTMME}}$	$\hat{\alpha }$	${\hat{\alpha }}_{M}$	$\hat{\alpha }\left(k\right)$	${\hat{\alpha }}_{M}\left(k\right)$	${\hat{\alpha }}_{\text{MRT}}$	${\hat{\alpha }}_{\text{RTMME}}$
0.3	1	0.7	43.418	1.975	30.622	1.590	28.798	1.532	43.418	1.975	30.622	1.590	26.378	1.454	43.418	1.975	30.622	1.590	24.277	1.386
		0.8	66.970	2.995	39.537	2.122	36.231	2.010	66.970	2.995	39.537	2.122	32.058	1.864	66.970	2.995	39.537	2.122	28.623	1.740
		0.9	143.790	6.312	52.339	3.194	45.398	2.921	143.790	6.312	52.339	3.194	37.510	2.594	143.790	6.312	52.339	3.194	31.684	2.338
		0.99	1790.873	76.051	36.821	5.131	29.299	4.271	1790.873	76.051	36.821	5.131	22.191	3.390	1790.873	76.051	36.821	5.131	17.718	2.798
	5	0.7	6.679	1.924	6.620	1.905	6.608	1.901	6.679	1.924	6.620	1.905	6.591	1.896	6.679	1.924	6.620	1.905	6.573	1.890
		0.8	9.507	2.838	9.383	2.793	9.358	2.784	9.507	2.838	9.383	2.793	9.322	2.771	9.507	2.838	9.383	2.793	9.285	2.758
		0.9	17.837	5.643	17.377	5.450	17.288	5.413	17.837	5.643	17.377	5.450	17.157	5.359	17.837	5.643	17.377	5.450	17.027	5.306
		0.99	177.299	63.383	138.858	43.305	133.290	40.828	177.299	63.383	138.858	43.305	125.773	37.648	177.299	63.383	138.858	43.305	119.087	34.970
	10	0.7	2.837	1.405	2.832	1.403	2.831	1.403	2.837	1.405	2.832	1.403	2.830	1.402	2.837	1.405	2.832	1.403	2.828	1.401
		0.8	4.370	2.150	4.358	2.144	4.355	2.143	4.370	2.150	4.358	2.144	4.352	2.141	4.370	2.150	4.358	2.144	4.348	2.139
		0.9	8.934	4.360	8.882	4.334	8.871	4.329	8.934	4.360	8.882	4.334	8.855	4.322	8.934	4.360	8.882	4.334	8.840	4.314
		0.99	103.740	48.970	96.088	45.343	94.699	44.685	103.740	48.970	96.088	45.343	92.695	43.735	103.740	48.970	96.088	45.343	90.779	42.827

0.7	1	0.7	43.418	1.975	21.166	1.281	18.955	1.204	43.418	1.975	21.166	1.281	16.297	1.108	43.418	1.975	21.166	1.281	14.218	1.029
		0.8	66.970	2.995	23.850	1.561	20.674	1.436	66.970	2.995	23.850	1.561	17.086	1.287	66.970	2.995	23.850	1.561	14.451	1.171
		0.9	143.790	6.312	24.476	1.998	20.200	1.778	143.790	6.312	24.476	1.998	15.822	1.534	143.790	6.312	24.476	1.998	12.891	1.355
		0.99	1790.873	76.051	12.903	2.127	10.354	1.760	1790.873	76.051	12.903	2.127	7.935	1.409	1790.873	76.051	12.903	2.127	6.406	1.189
	5	0.7	6.679	1.924	6.542	1.880	6.515	1.872	6.679	1.924	6.542	1.880	6.475	1.859	6.679	1.924	6.542	1.880	6.436	1.846
		0.8	9.507	2.838	9.221	2.736	9.166	2.716	9.507	2.838	9.221	2.736	9.085	2.687	9.507	2.838	9.221	2.736	9.005	2.659
		0.9	17.837	5.643	16.801	5.214	16.609	5.136	17.837	5.643	16.801	5.214	16.329	5.024	17.837	5.643	16.801	5.214	16.058	4.916
		0.99	177.299	63.383	108.816	31.115	101.158	28.426	177.299	63.383	108.816	31.115	91.433	25.213	177.299	63.383	108.816	31.115	83.324	22.686
	10	0.7	2.837	1.405	2.825	1.400	2.823	1.399	2.837	1.405	2.825	1.400	2.820	1.397	2.837	1.405	2.825	1.400	2.816	1.395
		0.8	4.370	2.150	4.342	2.136	4.336	2.134	4.370	2.150	4.342	2.136	4.328	2.130	4.370	2.150	4.342	2.136	4.320	2.126
		0.9	8.934	4.360	8.812	4.300	8.788	4.289	8.934	4.360	8.812	4.300	8.752	4.271	8.934	4.360	8.812	4.300	8.716	4.254
		0.99	103.740	48.970	87.575	41.308	84.965	40.070	103.740	48.970	87.575	41.308	81.351	38.357	103.740	48.970	87.575	41.308	78.056	36.795

0.9	1	0.7	2.837	1.405	2.822	1.398	2.819	1.397	43.418	1.975	18.124	1.174	13.463	1.000	43.418	1.975	18.124	1.174	11.571	0.924
		0.8	4.370	2.150	4.334	2.132	4.327	2.129	66.970	2.995	19.526	1.390	13.530	1.129	66.970	2.995	19.526	1.390	11.305	1.022
		0.9	8.934	4.360	8.778	4.284	8.747	4.269	143.790	6.312	18.748	1.700	11.919	1.292	143.790	6.312	18.748	1.700	9.681	1.139
		0.99	103.740	48.970	83.897	39.564	80.861	38.125	1790.873	76.051	9.532	1.641	5.913	1.119	1790.873	76.051	9.532	1.641	4.801	0.964
	5	0.7	6.679	1.924	6.504	1.868	6.470	1.857	6.679	1.924	6.504	1.868	6.419	1.841	6.679	1.924	6.504	1.868	6.369	1.825
		0.8	9.507	2.838	9.143	2.708	9.073	2.683	9.507	2.838	9.143	2.708	8.971	2.647	9.507	2.838	9.143	2.708	8.870	2.612
		0.9	17.837	5.643	16.528	5.103	16.289	5.008	17.837	5.643	16.528	5.103	15.944	4.872	17.837	5.643	16.528	5.103	15.613	4.743
		0.99	177.299	63.383	98.186	27.421	90.186	24.816	177.299	63.383	98.186	27.421	80.244	21.759	177.299	63.383	98.186	27.421	72.146	19.393
	10	0.7	2.837	1.405	2.822	1.398	2.819	1.397	2.837	1.405	2.822	1.398	2.815	1.395	2.837	1.405	2.822	1.398	2.810	1.393
		0.8	4.370	2.150	4.334	2.132	4.327	2.129	4.370	2.150	4.334	2.132	4.316	2.124	4.370	2.150	4.334	2.132	4.306	2.119
		0.9	8.934	4.360	8.778	4.284	8.747	4.269	8.934	4.360	8.778	4.284	8.701	4.246	8.934	4.360	8.778	4.284	8.656	4.224
		0.99	103.740	48.970	83.897	39.564	80.861	38.125	103.740	48.970	83.897	39.564	76.731	36.167	103.740	48.970	83.897	39.564	73.036	34.415

**Table 12:** Estimated MSEs of OLS, MRE, MRE, MRT, and RTMME at *n* = 20 and *p* = 7 when there are two outliers.

*k*	Sigma	Rho	*d* = 0.2						*d* = 0.5						*d* = 0.8					
			$\hat{\alpha }$	${\hat{\alpha }}_{M}$	$\hat{\alpha }\left(k\right)$	${\hat{\alpha }}_{M}\left(k\right)$	${\hat{\alpha }}_{\text{MRT}}$	${\hat{\alpha }}_{\text{RTMME}}$	$\hat{\alpha }$	${\hat{\alpha }}_{M}$	$\hat{\alpha }\left(k\right)$	${\hat{\alpha }}_{M}\left(k\right)$	${\hat{\alpha }}_{\text{MRT}}$	${\hat{\alpha }}_{\text{RTMME}}$	$\hat{\alpha }$	${\hat{\alpha }}_{M}$	$\hat{\alpha }\left(k\right)$	${\hat{\alpha }}_{M}\left(k\right)$	${\hat{\alpha }}_{\text{MRT}}$	${\hat{\alpha }}_{\text{RTMME}}$
0.3	1	0.7	105.945	3.884	78.646	3.162	74.687	3.052	105.945	3.884	78.646	3.162	69.399	2.902	105.945	3.884	78.646	3.162	64.768	2.768
		0.8	156.521	5.766	100.774	4.205	93.860	3.998	156.521	5.766	100.774	4.205	85.027	3.727	156.521	5.766	100.774	4.205	77.650	3.494
		0.9	315.660	11.802	140.734	6.437	126.344	5.937	315.660	11.802	140.734	6.437	109.484	5.329	315.660	11.802	140.734	6.437	96.547	4.843
		0.99	3440.840	126.654	194.473	11.626	157.663	9.599	3440.840	126.654	194.473	11.626	120.114	7.484	3440.840	126.654	194.473	11.626	94.981	6.041
	5	0.7	12.325	2.265	12.218	2.243	12.197	2.238	12.325	2.265	12.218	2.243	12.166	2.232	12.325	2.265	12.218	2.243	12.134	2.225
		0.8	17.632	3.304	17.412	3.254	17.368	3.244	17.632	3.304	17.412	3.254	17.303	3.230	17.632	3.304	17.412	3.254	17.239	3.215
		0.9	33.218	6.515	32.413	6.304	32.257	6.264	33.218	6.515	32.413	6.304	32.025	6.204	33.218	6.515	32.413	6.304	31.797	6.145
		0.99	327.375	71.474	261.240	50.147	251.257	47.452	327.375	71.474	261.240	50.147	237.627	43.963	327.375	71.474	261.240	50.147	225.359	40.997
	10	0.7	7.152	3.480	7.139	3.474	7.137	3.473	7.152	3.480	7.139	3.474	7.133	3.471	7.152	3.480	7.139	3.474	7.129	3.470
		0.8	10.655	5.157	10.626	5.144	10.621	5.141	10.655	5.157	10.626	5.144	10.612	5.137	10.655	5.157	10.626	5.144	10.603	5.133
		0.9	21.143	10.222	21.022	10.165	20.998	10.154	21.143	10.222	21.022	10.165	20.962	10.137	21.143	10.222	21.022	10.165	20.926	10.121
		0.99	222.411	106.264	207.282	99.298	204.530	98.029	222.411	106.264	207.282	99.298	200.553	96.195	222.411	106.264	207.282	99.298	196.749	94.439

0.7	1	0.7	105.945	3.884	57.827	2.562	52.814	2.408	105.945	3.884	57.827	2.562	46.673	2.212	105.945	3.884	57.827	2.562	41.757	2.049
		0.8	156.521	5.766	67.178	3.150	60.021	2.906	156.521	5.766	67.178	3.150	51.678	2.608	156.521	5.766	67.178	3.150	45.309	2.369
		0.9	315.660	11.802	79.699	4.177	69.053	3.734	315.660	11.802	79.699	4.177	57.385	3.224	315.660	11.802	79.699	4.177	48.936	2.837
		0.99	3440.840	126.654	66.720	4.388	51.399	3.478	3440.840	126.654	66.720	4.388	36.861	2.605	3440.840	126.654	66.720	4.388	27.825	2.059
	5	0.7	12.325	2.265	12.079	2.214	12.031	2.204	12.325	2.265	12.079	2.214	11.959	2.190	12.325	2.265	12.079	2.214	11.889	2.175
		0.8	17.632	3.304	17.126	3.190	17.028	3.168	17.632	3.304	17.126	3.190	16.883	3.136	17.632	3.304	17.126	3.190	16.740	3.104
		0.9	33.218	6.515	31.399	6.044	31.058	5.958	33.218	6.515	31.399	6.044	30.560	5.834	33.218	6.515	31.399	6.044	30.078	5.715
		0.99	327.375	71.474	206.265	36.676	191.848	33.624	327.375	71.474	206.265	36.676	173.336	29.931	327.375	71.474	206.265	36.676	157.747	26.990
	10	0.7	7.152	3.480	7.122	3.466	7.117	3.464	7.152	3.480	7.122	3.466	7.108	3.460	7.152	3.480	7.122	3.466	7.099	3.455
		0.8	10.655	5.157	10.588	5.126	10.575	5.119	10.655	5.157	10.588	5.126	10.555	5.110	10.655	5.157	10.588	5.126	10.535	5.101
		0.9	21.143	10.222	20.863	10.091	20.808	10.065	21.143	10.222	20.863	10.091	20.726	10.027	21.143	10.222	20.863	10.091	20.644	9.989
		0.99	222.411	106.264	190.376	91.494	185.175	89.089	222.411	106.264	190.376	91.494	177.958	85.746	222.411	106.264	190.376	91.494	171.360	82.685

0.9	1	0.7	7.152	3.480	7.114	3.463	7.107	3.459	105.945	3.884	50.909	2.348	39.940	1.987	105.945	3.884	50.909	2.348	35.293	1.824
		0.8	10.655	5.157	10.569	5.117	10.552	5.109	156.521	5.766	57.386	2.813	43.019	2.281	156.521	5.766	57.386	2.813	37.308	2.054
		0.9	21.143	10.222	20.784	10.054	20.714	10.022	315.660	11.802	65.286	3.572	45.984	2.698	315.660	11.802	65.286	3.572	38.802	2.352
		0.99	222.411	106.264	183.045	88.102	176.979	85.292	3440.840	126.654	46.447	3.182	24.966	1.885	3440.840	126.654	46.447	3.182	18.663	1.503
	5	0.7	12.325	2.265	12.010	2.200	11.949	2.188	12.325	2.265	12.010	2.200	11.858	2.169	12.325	2.265	12.010	2.200	11.769	2.151
		0.8	17.632	3.304	16.986	3.159	16.862	3.131	17.632	3.304	16.986	3.159	16.679	3.091	17.632	3.304	16.986	3.159	16.500	3.051
		0.9	33.218	6.515	30.914	5.922	30.491	5.816	33.218	6.515	30.914	5.922	29.876	5.665	33.218	6.515	30.914	5.922	29.284	5.522
		0.99	327.375	71.474	186.213	32.474	170.948	29.471	327.375	71.474	186.213	32.474	151.794	25.902	327.375	71.474	186.213	32.474	136.061	23.107
	10	0.7	7.152	3.480	7.114	3.463	7.107	3.459	7.152	3.480	7.114	3.463	7.095	3.454	7.152	3.480	7.114	3.463	7.084	3.448
		0.8	10.655	5.157	10.569	5.117	10.552	5.109	10.655	5.157	10.569	5.117	10.526	5.097	10.655	5.157	10.569	5.117	10.501	5.085
		0.9	21.143	10.222	20.784	10.054	20.714	10.022	21.143	10.222	20.784	10.054	20.610	9.973	21.143	10.222	20.784	10.054	20.506	9.925
		0.99	222.411	106.264	183.045	88.102	176.979	85.292	222.411	106.264	183.045	88.102	168.702	81.451	222.411	106.264	183.045	88.102	161.274	77.996

**Table 13:** Estimated MSEs of OLS, MRE, MRE, MRT, and RTMME at *n* = 50 and *p* = 7 when there is no outlier.

*k*	Sigma	Rho	*d* = 0.2						*d* = 0.5						*d* = 0.8					
			$\hat{\alpha }$	${\hat{\alpha }}_{M}$	$\hat{\alpha }\left(k\right)$	${\hat{\alpha }}_{M}\left(k\right)$	${\hat{\alpha }}_{\text{MRT}}$	${\hat{\alpha }}_{\text{RTMME}}$	$\hat{\alpha }$	${\hat{\alpha }}_{M}$	$\hat{\alpha }\left(k\right)$	${\hat{\alpha }}_{M}\left(k\right)$	${\hat{\alpha }}_{\text{MRT}}$	${\hat{\alpha }}_{\text{RTMME}}$	$\hat{\alpha }$	${\hat{\alpha }}_{M}$	$\hat{\alpha }\left(k\right)$	${\hat{\alpha }}_{M}\left(k\right)$	${\hat{\alpha }}_{\text{MRT}}$	${\hat{\alpha }}_{\text{RTMME}}$
0.3	1	0.7	0.355	0.379	0.337	0.360	0.334	0.356	0.355	0.379	0.337	0.360	0.329	0.351	0.355	0.379	0.337	0.360	0.325	0.346
		0.8	0.556	0.593	0.513	0.546	0.506	0.538	0.556	0.593	0.513	0.546	0.495	0.527	0.556	0.593	0.513	0.546	0.485	0.516
		0.9	1.188	1.272	0.980	1.049	0.948	1.015	1.188	1.272	0.980	1.049	0.905	0.968	1.188	1.272	0.980	1.049	0.866	0.927
		0.99	14.115	14.999	3.778	3.995	3.350	3.542	14.115	14.999	3.778	3.995	2.865	3.026	14.115	14.999	3.778	3.995	2.500	2.639
	5	0.7	0.365	0.383	0.364	0.382	0.364	0.382	0.365	0.383	0.364	0.382	0.364	0.382	0.365	0.383	0.364	0.382	0.364	0.381
		0.8	0.542	0.574	0.540	0.572	0.539	0.571	0.542	0.574	0.540	0.572	0.539	0.571	0.542	0.574	0.540	0.572	0.538	0.570
		0.9	1.112	1.186	1.103	1.176	1.101	1.174	1.112	1.186	1.103	1.176	1.098	1.171	1.112	1.186	1.103	1.176	1.095	1.168
		0.99	13.448	14.327	11.941	12.718	11.682	12.441	13.448	14.327	11.941	12.718	11.315	12.049	13.448	14.327	11.941	12.718	10.973	11.683
	10	0.7	0.320	0.339	0.320	0.339	0.320	0.339	0.320	0.339	0.320	0.339	0.320	0.339	0.320	0.339	0.320	0.339	0.320	0.339
		0.8	0.490	0.515	0.489	0.515	0.489	0.515	0.490	0.515	0.489	0.515	0.489	0.515	0.490	0.515	0.489	0.515	0.489	0.514
		0.9	1.022	1.083	1.020	1.081	1.020	1.081	1.022	1.083	1.020	1.081	1.020	1.080	1.022	1.083	1.020	1.081	1.019	1.080
		0.99	12.424	13.112	12.100	12.773	12.038	12.707	12.424	13.112	12.100	12.773	11.945	12.610	12.424	13.112	12.100	12.773	11.854	12.514

0.7	1	0.7	0.355	0.379	0.318	0.338	0.311	0.332	0.355	0.379	0.318	0.338	0.303	0.323	0.355	0.379	0.318	0.338	0.296	0.315
		0.8	0.556	0.593	0.468	0.498	0.455	0.484	0.556	0.593	0.468	0.498	0.437	0.465	0.556	0.593	0.468	0.498	0.422	0.448
		0.9	1.188	1.272	0.807	0.863	0.764	0.816	1.188	1.272	0.807	0.863	0.709	0.757	1.188	1.272	0.807	0.863	0.664	0.708
		0.99	14.115	14.999	2.033	2.144	1.744	1.837	14.115	14.999	2.033	2.144	1.437	1.511	14.115	14.999	2.033	2.144	1.224	1.284
	5	0.7	0.365	0.383	0.363	0.381	0.363	0.381	0.365	0.383	0.363	0.381	0.362	0.380	0.365	0.383	0.363	0.381	0.362	0.379
		0.8	0.542	0.574	0.537	0.569	0.536	0.568	0.542	0.574	0.537	0.569	0.535	0.567	0.542	0.574	0.537	0.569	0.534	0.565
		0.9	1.112	1.186	1.091	1.163	1.086	1.159	1.112	1.186	1.091	1.163	1.080	1.152	1.112	1.186	1.091	1.163	1.074	1.146
		0.99	13.448	14.327	10.416	11.089	9.978	10.621	13.448	14.327	10.416	11.089	9.393	9.995	13.448	14.327	10.416	11.089	8.880	9.447
	10	0.7	0.320	0.339	0.320	0.339	0.320	0.338	0.320	0.339	0.320	0.339	0.320	0.338	0.320	0.339	0.320	0.339	0.320	0.338
		0.8	0.490	0.515	0.489	0.514	0.489	0.514	0.490	0.515	0.489	0.514	0.488	0.514	0.490	0.515	0.489	0.514	0.488	0.514
		0.9	1.022	1.083	1.018	1.079	1.017	1.078	1.022	1.083	1.018	1.079	1.016	1.077	1.022	1.083	1.018	1.079	1.015	1.075
		0.99	12.424	13.112	11.695	12.348	11.560	12.206	12.424	13.112	11.695	12.348	11.363	12.000	12.424	13.112	11.695	12.348	11.173	11.801

0.9	1	0.7	0.355	0.379	0.309	0.329	0.302	0.322	0.355	0.379	0.309	0.329	0.293	0.311	0.355	0.379	0.309	0.329	0.284	0.302
		0.8	0.556	0.593	0.450	0.478	0.435	0.462	0.556	0.593	0.450	0.478	0.416	0.441	0.556	0.593	0.450	0.478	0.399	0.423
		0.9	1.188	1.272	0.747	0.798	0.702	0.749	1.188	1.272	0.747	0.798	0.647	0.689	1.188	1.272	0.747	0.798	0.602	0.642
		0.99	14.115	14.999	1.644	1.731	1.402	1.473	14.115	14.999	1.644	1.731	1.152	1.208	14.115	14.999	1.644	1.731	0.986	1.030
	5	0.7	0.365	0.383	0.363	0.380	0.362	0.380	0.365	0.383	0.363	0.380	0.362	0.379	0.365	0.383	0.363	0.380	0.361	0.378
		0.8	0.542	0.574	0.536	0.568	0.535	0.566	0.542	0.574	0.536	0.568	0.533	0.565	0.542	0.574	0.536	0.568	0.532	0.563
		0.9	1.112	1.186	1.085	1.157	1.079	1.151	1.112	1.186	1.085	1.157	1.072	1.143	1.112	1.186	1.085	1.157	1.064	1.135
		0.99	13.448	14.327	9.803	10.433	9.316	9.913	13.448	14.327	9.803	10.433	8.679	9.233	13.448	14.327	9.803	10.433	8.134	8.651
	10	0.7	0.320	0.339	0.320	0.338	0.320	0.338	0.320	0.339	0.320	0.338	0.320	0.338	0.320	0.339	0.320	0.338	0.319	0.338
		0.8	0.490	0.515	0.488	0.514	0.488	0.514	0.490	0.515	0.488	0.514	0.488	0.513	0.490	0.515	0.488	0.514	0.488	0.513
		0.9	1.022	1.083	1.017	1.078	1.016	1.077	1.022	1.083	1.017	1.078	1.014	1.075	1.022	1.083	1.017	1.078	1.013	1.073
		0.99	12.424	13.112	11.503	12.147	11.336	11.971	12.424	13.112	11.503	12.147	11.094	11.718	12.424	13.112	11.503	12.147	10.863	11.476

**Table 14:** Estimated MSEs of OLS, MRE, MRE, MRT, and RTMME at *n* = 50 and *p* = 7 when there is one outlier.

*k*	Sigma	Rho	*d* = 0.2						*d* = 0.5						*d* = 0.8					
			$\hat{\alpha }$	${\hat{\alpha }}_{M}$	$\hat{\alpha }\left(k\right)$	${\hat{\alpha }}_{M}\left(k\right)$	${\hat{\alpha }}_{\text{MRT}}$	${\hat{\alpha }}_{\text{RTMME}}$	$\hat{\alpha }$	${\hat{\alpha }}_{M}$	$\hat{\alpha }\left(k\right)$	${\hat{\alpha }}_{M}\left(k\right)$	${\hat{\alpha }}_{\text{MRT}}$	${\hat{\alpha }}_{\text{RTMME}}$	$\hat{\alpha }$	${\hat{\alpha }}_{M}$	$\hat{\alpha }\left(k\right)$	${\hat{\alpha }}_{M}\left(k\right)$	${\hat{\alpha }}_{\text{MRT}}$	${\hat{\alpha }}_{\text{RTMME}}$
0.3	1	0.7	2.368	0.394	2.266	0.377	2.246	0.374	2.368	0.394	2.266	0.377	2.218	0.370	2.368	0.394	2.266	0.377	2.191	0.365
		0.8	3.716	0.626	3.435	0.582	3.384	0.574	3.716	0.626	3.435	0.582	3.311	0.563	3.716	0.626	3.435	0.582	3.241	0.552
		0.9	7.504	1.319	6.208	1.102	6.004	1.068	7.504	1.319	6.208	1.102	5.725	1.023	7.504	1.319	6.208	1.102	5.472	0.982
		0.99	88.326	15.690	21.517	4.214	18.804	3.735	88.326	15.690	21.517	4.214	15.750	3.191	88.326	15.690	21.517	4.214	13.476	2.781
	5	0.7	0.841	0.410	0.840	0.410	0.840	0.409	0.841	0.410	0.840	0.410	0.840	0.409	0.841	0.410	0.840	0.410	0.839	0.409
		0.8	1.296	0.623	1.293	0.621	1.293	0.621	1.296	0.623	1.293	0.621	1.292	0.620	1.296	0.623	1.293	0.621	1.291	0.620
		0.9	2.784	1.281	2.768	1.271	2.764	1.269	2.784	1.281	2.768	1.271	2.760	1.267	2.784	1.281	2.768	1.271	2.755	1.264
		0.99	38.788	15.976	34.689	14.224	33.980	13.922	38.788	15.976	34.689	14.224	32.973	13.494	38.788	15.976	34.689	14.224	32.029	13.094
	10	0.7	0.428	0.347	0.428	0.347	0.428	0.347	0.428	0.347	0.428	0.347	0.428	0.346	0.428	0.347	0.428	0.347	0.428	0.346
		0.8	0.669	0.529	0.669	0.529	0.669	0.529	0.669	0.529	0.669	0.529	0.669	0.529	0.669	0.529	0.669	0.529	0.668	0.529
		0.9	1.399	1.111	1.397	1.109	1.397	1.109	1.399	1.111	1.397	1.109	1.396	1.108	1.399	1.111	1.397	1.109	1.395	1.108
		0.99	17.302	13.500	16.828	13.151	16.737	13.084	17.302	13.500	16.828	13.151	16.601	12.984	17.302	13.500	16.828	13.151	16.468	12.886

0.7	1	0.7	2.368	0.394	2.144	0.358	2.104	0.352	2.368	0.394	2.144	0.358	2.048	0.344	2.368	0.394	2.144	0.358	1.995	0.336
		0.8	3.716	0.626	3.125	0.535	3.030	0.521	3.716	0.626	3.125	0.535	2.900	0.503	3.716	0.626	3.125	0.535	2.781	0.486
		0.9	7.504	1.319	5.078	0.918	4.780	0.871	7.504	1.319	5.078	0.918	4.397	0.810	7.504	1.319	5.078	0.918	4.075	0.761
		0.99	88.326	15.690	10.595	2.257	8.826	1.933	88.326	15.690	10.595	2.257	6.951	1.587	88.326	15.690	10.595	2.257	5.654	1.347
	5	0.7	0.841	0.410	0.839	0.409	0.838	0.408	0.841	0.410	0.839	0.409	0.838	0.408	0.841	0.410	0.839	0.409	0.837	0.407
		0.8	1.296	0.623	1.290	0.619	1.288	0.618	1.296	0.623	1.290	0.619	1.286	0.617	1.296	0.623	1.290	0.619	1.284	0.615
		0.9	2.784	1.281	2.746	1.259	2.739	1.254	2.784	1.281	2.746	1.259	2.727	1.248	2.784	1.281	2.746	1.259	2.716	1.242
		0.99	38.788	15.976	30.492	12.443	29.275	11.929	38.788	15.976	30.492	12.443	27.640	11.241	38.788	15.976	30.492	12.443	26.197	10.636
	10	0.7	0.428	0.347	0.428	0.346	0.427	0.346	0.428	0.347	0.428	0.346	0.427	0.346	0.428	0.347	0.428	0.346	0.427	0.346
		0.8	0.669	0.529	0.668	0.529	0.668	0.528	0.669	0.529	0.668	0.529	0.668	0.528	0.669	0.529	0.668	0.529	0.668	0.528
		0.9	1.399	1.111	1.394	1.107	1.393	1.106	1.399	1.111	1.394	1.107	1.392	1.105	1.399	1.111	1.394	1.107	1.390	1.103
		0.99	17.302	13.500	16.236	12.715	16.039	12.569	17.302	13.500	16.236	12.715	15.751	12.357	17.302	13.500	16.236	12.715	15.474	12.153

0.9	1	0.7	2.368	0.394	2.088	0.350	2.040	0.343	2.368	0.394	2.088	0.350	1.974	0.333	2.368	0.394	2.088	0.350	1.911	0.325
		0.8	3.716	0.626	2.992	0.516	2.882	0.500	3.716	0.626	2.992	0.516	2.733	0.479	3.716	0.626	2.992	0.516	2.599	0.461
		0.9	7.504	1.319	4.663	0.852	4.348	0.803	7.504	1.319	4.663	0.852	3.952	0.742	7.504	1.319	4.663	0.852	3.625	0.692
		0.99	88.326	15.690	8.212	1.820	6.737	1.548	88.326	15.690	8.212	1.820	5.216	1.266	88.326	15.690	8.212	1.820	4.192	1.078
	5	0.7	0.841	0.410	0.838	0.408	0.838	0.408	0.841	0.410	0.838	0.408	0.837	0.407	0.841	0.410	0.838	0.408	0.836	0.406
		0.8	1.296	0.623	1.288	0.618	1.286	0.617	1.296	0.623	1.288	0.618	1.284	0.615	1.296	0.623	1.288	0.618	1.281	0.613
		0.9	2.784	1.281	2.735	1.253	2.726	1.247	2.784	1.281	2.735	1.253	2.712	1.239	2.784	1.281	2.735	1.253	2.698	1.231
		0.99	38.788	15.976	28.786	11.723	27.423	11.150	38.788	15.976	28.786	11.723	25.630	10.398	38.788	15.976	28.786	11.723	24.081	9.753
	10	0.7	0.428	0.347	0.427	0.346	0.427	0.346	0.428	0.347	0.427	0.346	0.427	0.346	0.428	0.347	0.427	0.346	0.427	0.346
		0.8	0.669	0.529	0.668	0.528	0.668	0.528	0.669	0.529	0.668	0.528	0.667	0.528	0.669	0.529	0.668	0.528	0.667	0.528
		0.9	1.399	1.111	1.393	1.106	1.392	1.104	1.399	1.111	1.393	1.106	1.390	1.103	1.399	1.111	1.393	1.106	1.388	1.101
		0.99	17.302	13.500	15.955	12.508	15.711	12.327	17.302	13.500	15.955	12.508	15.359	12.067	17.302	13.500	15.955	12.508	15.022	11.818

**Table 15:** Estimated MSEs of OLS, MRE, MRE, MRT, and RTMME at *n* = 50 and *p* = 7 when there are two outliers.

*k*	Sigma	Rho	*d* = 0.2						*d* = 0.5						*d* = 0.8					
			$\hat{\alpha }$	${\hat{\alpha }}_{M}$	$\hat{\alpha }\left(k\right)$	${\hat{\alpha }}_{M}\left(k\right)$	${\hat{\alpha }}_{\text{MRT}}$	${\hat{\alpha }}_{\text{RTMME}}$	$\hat{\alpha }$	${\hat{\alpha }}_{M}$	$\hat{\alpha }\left(k\right)$	${\hat{\alpha }}_{M}\left(k\right)$	${\hat{\alpha }}_{\text{MRT}}$	${\hat{\alpha }}_{\text{RTMME}}$	$\hat{\alpha }$	${\hat{\alpha }}_{M}$	$\hat{\alpha }\left(k\right)$	${\hat{\alpha }}_{M}\left(k\right)$	${\hat{\alpha }}_{\text{MRT}}$	${\hat{\alpha }}_{\text{RTMME}}$
0.3	1	0.7	3.708	0.423	3.546	0.399	3.515	0.394	3.708	0.423	3.546	0.399	3.470	0.388	3.708	0.423	3.546	0.399	3.426	0.382
		0.8	5.409	0.657	5.051	0.600	4.986	0.591	5.409	0.657	5.051	0.600	4.891	0.576	5.409	0.657	5.051	0.600	4.800	0.563
		0.9	10.810	1.399	9.329	1.140	9.089	1.101	10.810	1.399	9.329	1.140	8.754	1.047	10.810	1.399	9.329	1.140	8.446	0.999
		0.99	113.434	16.400	42.585	4.289	37.921	3.788	113.434	16.400	42.585	4.289	32.335	3.219	113.434	16.400	42.585	4.289	27.948	2.791
	5	0.7	1.359	0.435	1.357	0.434	1.357	0.434	1.359	0.435	1.357	0.434	1.356	0.434	1.359	0.435	1.357	0.434	1.356	0.434
		0.8	2.109	0.665	2.104	0.663	2.103	0.663	2.109	0.665	2.104	0.663	2.102	0.662	2.109	0.665	2.104	0.663	2.101	0.662
		0.9	4.488	1.361	4.462	1.351	4.457	1.349	4.488	1.361	4.462	1.351	4.449	1.346	4.488	1.361	4.462	1.351	4.441	1.343
		0.99	59.623	17.056	53.650	15.213	52.613	14.894	59.623	17.056	53.650	15.213	51.140	14.443	59.623	17.056	53.650	15.213	49.758	14.020
	10	0.7	0.630	0.372	0.630	0.372	0.630	0.372	0.630	0.372	0.630	0.372	0.629	0.372	0.630	0.372	0.630	0.372	0.629	0.372
		0.8	0.975	0.567	0.975	0.567	0.975	0.567	0.975	0.567	0.975	0.567	0.974	0.567	0.975	0.567	0.975	0.567	0.974	0.567
		0.9	2.016	1.184	2.014	1.182	2.013	1.182	2.016	1.184	2.014	1.182	2.013	1.181	2.016	1.184	2.014	1.182	2.012	1.181
		0.99	24.543	14.279	23.987	13.924	23.880	13.856	24.543	14.279	23.987	13.924	23.721	13.754	24.543	14.279	23.987	13.924	23.564	13.654

0.7	1	0.7	3.708	0.423	3.351	0.371	3.288	0.363	3.708	0.423	3.351	0.371	3.197	0.351	3.708	0.423	3.351	0.371	3.112	0.340
		0.8	5.409	0.657	4.647	0.541	4.522	0.524	5.409	0.657	4.647	0.541	4.346	0.500	5.409	0.657	4.647	0.541	4.184	0.479
		0.9	10.810	1.399	7.955	0.926	7.575	0.871	10.810	1.399	7.955	0.926	7.071	0.803	10.810	1.399	7.955	0.926	6.631	0.746
		0.99	113.434	16.400	22.122	2.244	18.411	1.906	113.434	16.400	22.122	2.244	14.378	1.547	113.434	16.400	22.122	2.244	11.534	1.298
	5	0.7	1.359	0.435	1.355	0.433	1.354	0.433	1.359	0.435	1.355	0.433	1.353	0.433	1.359	0.435	1.355	0.433	1.352	0.432
		0.8	2.109	0.665	2.098	0.661	2.096	0.660	2.109	0.665	2.098	0.661	2.093	0.658	2.109	0.665	2.098	0.661	2.090	0.657
		0.9	4.488	1.361	4.428	1.338	4.416	1.333	4.488	1.361	4.428	1.338	4.398	1.327	4.488	1.361	4.428	1.338	4.381	1.320
		0.99	59.623	17.056	47.501	13.333	45.711	12.790	59.623	17.056	47.501	13.333	43.298	12.061	59.623	17.056	47.501	13.333	41.162	11.420
	10	0.7	0.630	0.372	0.629	0.372	0.629	0.372	0.630	0.372	0.629	0.372	0.629	0.372	0.630	0.372	0.629	0.372	0.629	0.371
		0.8	0.975	0.567	0.974	0.567	0.974	0.566	0.975	0.567	0.974	0.567	0.974	0.566	0.975	0.567	0.974	0.567	0.973	0.566
		0.9	2.016	1.184	2.011	1.180	2.010	1.179	2.016	1.184	2.011	1.180	2.008	1.178	2.016	1.184	2.011	1.180	2.006	1.176
		0.99	24.543	14.279	23.291	13.480	23.058	13.332	24.543	14.279	23.291	13.480	22.718	13.116	24.543	14.279	23.291	13.480	22.390	12.907

0.9	1	0.7	3.708	0.423	3.261	0.359	3.185	0.349	3.708	0.423	3.261	0.359	3.076	0.336	3.708	0.423	3.261	0.359	2.975	0.324
		0.8	5.409	0.657	4.470	0.517	4.322	0.497	5.409	0.657	4.470	0.517	4.119	0.471	5.409	0.657	4.470	0.517	3.933	0.449
		0.9	10.810	1.399	7.423	0.850	7.004	0.794	10.810	1.399	7.423	0.850	6.459	0.725	10.810	1.399	7.423	0.850	5.991	0.670
		0.99	113.434	16.400	17.101	1.788	13.911	1.506	113.434	16.400	17.101	1.788	10.566	1.215	113.434	16.400	17.101	1.788	8.292	1.022
	5	0.7	1.359	0.435	1.354	0.433	1.353	0.432	1.359	0.435	1.354	0.433	1.351	0.432	1.359	0.435	1.354	0.433	1.350	0.431
		0.8	2.109	0.665	2.095	0.659	2.093	0.658	2.109	0.665	2.095	0.659	2.089	0.657	2.109	0.665	2.095	0.659	2.085	0.655
		0.9	4.488	1.361	4.411	1.331	4.396	1.326	4.488	1.361	4.411	1.331	4.373	1.317	4.488	1.361	4.411	1.331	4.351	1.309
		0.99	59.623	17.056	44.990	12.572	42.977	11.965	59.623	17.056	44.990	12.572	40.319	11.168	59.623	17.056	44.990	12.572	38.014	10.483
	10	0.7	0.630	0.372	0.629	0.372	0.629	0.372	0.630	0.372	0.629	0.372	0.629	0.371	0.630	0.372	0.629	0.372	0.629	0.371
		0.8	0.975	0.567	0.974	0.566	0.974	0.566	0.975	0.567	0.974	0.566	0.973	0.566	0.975	0.567	0.974	0.566	0.973	0.566
		0.9	2.016	1.184	2.009	1.178	2.008	1.177	2.016	1.184	2.009	1.178	2.006	1.176	2.016	1.184	2.009	1.178	2.003	1.174
		0.99	24.543	14.279	22.960	13.269	22.671	13.085	24.543	14.279	22.960	13.269	22.253	12.820	24.543	14.279	22.960	13.269	21.853	12.567

**Table 16:** Estimated MSEs of OLS, MRE, MRE, MRT, and RTMME at *n* = 100 and *p* = 7 when there is no outlier.

*k*	Sigma	Rho	*d* = 0.2						*d* = 0.5						*d* = 0.8					
			$\hat{\alpha }$	${\hat{\alpha }}_{M}$	$\hat{\alpha }\left(k\right)$	${\hat{\alpha }}_{M}\left(k\right)$	${\hat{\alpha }}_{\text{MRT}}$	${\hat{\alpha }}_{\text{RTMME}}$	$\hat{\alpha }$	${\hat{\alpha }}_{M}$	$\hat{\alpha }\left(k\right)$	${\hat{\alpha }}_{M}\left(k\right)$	${\hat{\alpha }}_{\text{MRT}}$	${\hat{\alpha }}_{\text{RTMME}}$	$\hat{\alpha }$	${\hat{\alpha }}_{M}$	$\hat{\alpha }\left(k\right)$	${\hat{\alpha }}_{M}\left(k\right)$	${\hat{\alpha }}_{\text{MRT}}$	${\hat{\alpha }}_{\text{RTMME}}$
0.3	1	0.7	0.175	0.185	0.171	0.182	0.171	0.181	0.175	0.185	0.171	0.182	0.170	0.180	0.175	0.185	0.171	0.182	0.169	0.179
		0.8	0.276	0.293	0.267	0.283	0.265	0.281	0.276	0.293	0.267	0.283	0.263	0.278	0.276	0.293	0.267	0.283	0.260	0.276
		0.9	0.543	0.573	0.501	0.529	0.494	0.521	0.543	0.573	0.501	0.529	0.483	0.510	0.543	0.573	0.501	0.529	0.473	0.499
		0.99	6.136	6.416	2.909	3.055	2.653	2.787	6.136	6.416	2.909	3.055	2.348	2.468	6.136	6.416	2.909	3.055	2.110	2.218
	5	0.7	0.150	0.158	0.150	0.158	0.150	0.158	0.150	0.158	0.150	0.158	0.150	0.158	0.150	0.158	0.150	0.158	0.150	0.158
		0.8	0.229	0.242	0.229	0.241	0.229	0.241	0.229	0.242	0.229	0.241	0.229	0.241	0.229	0.242	0.229	0.241	0.229	0.241
		0.9	0.495	0.524	0.494	0.523	0.493	0.522	0.495	0.524	0.494	0.523	0.493	0.522	0.495	0.524	0.494	0.523	0.492	0.521
		0.99	5.646	5.955	5.376	5.669	5.325	5.615	5.646	5.955	5.376	5.669	5.250	5.537	5.646	5.955	5.376	5.669	5.178	5.461
	10	0.7	0.156	0.164	0.156	0.164	0.156	0.164	0.156	0.164	0.156	0.164	0.156	0.164	0.156	0.164	0.156	0.164	0.156	0.164
		0.8	0.229	0.243	0.229	0.243	0.229	0.243	0.229	0.243	0.229	0.243	0.229	0.243	0.229	0.243	0.229	0.243	0.229	0.243
		0.9	0.457	0.480	0.456	0.480	0.456	0.480	0.457	0.480	0.456	0.480	0.456	0.480	0.457	0.480	0.456	0.480	0.456	0.479
		0.99	5.366	5.735	5.308	5.672	5.296	5.660	5.366	5.735	5.308	5.672	5.279	5.641	5.366	5.735	5.308	5.672	5.262	5.623

0.7	1	0.7	0.175	0.185	0.167	0.177	0.165	0.175	0.175	0.185	0.167	0.177	0.163	0.173	0.175	0.185	0.167	0.177	0.162	0.171
		0.8	0.276	0.293	0.256	0.271	0.253	0.268	0.276	0.293	0.256	0.271	0.248	0.262	0.276	0.293	0.256	0.271	0.243	0.257
		0.9	0.543	0.573	0.456	0.482	0.443	0.467	0.543	0.573	0.456	0.482	0.425	0.448	0.543	0.573	0.456	0.482	0.409	0.431
		0.99	6.136	6.416	1.793	1.884	1.587	1.667	6.136	6.416	1.793	1.884	1.356	1.424	6.136	6.416	1.793	1.884	1.188	1.246
	5	0.7	0.150	0.158	0.149	0.158	0.149	0.158	0.150	0.158	0.149	0.158	0.149	0.158	0.150	0.158	0.149	0.158	0.149	0.158
		0.8	0.229	0.242	0.228	0.241	0.228	0.241	0.229	0.242	0.228	0.241	0.228	0.241	0.229	0.242	0.228	0.241	0.228	0.240
		0.9	0.495	0.524	0.491	0.520	0.491	0.519	0.495	0.524	0.491	0.520	0.490	0.518	0.495	0.524	0.491	0.520	0.488	0.517
		0.99	5.646	5.955	5.056	5.331	4.953	5.223	5.646	5.955	5.056	5.331	4.808	5.070	5.646	5.955	5.056	5.331	4.672	4.927
	10	0.7	0.156	0.164	0.156	0.164	0.156	0.164	0.156	0.164	0.156	0.164	0.155	0.163	0.156	0.164	0.156	0.164	0.155	0.163
		0.8	0.229	0.243	0.229	0.243	0.229	0.243	0.229	0.243	0.229	0.243	0.229	0.243	0.229	0.243	0.229	0.243	0.229	0.243
		0.9	0.457	0.480	0.456	0.479	0.456	0.479	0.457	0.480	0.456	0.479	0.455	0.479	0.457	0.480	0.456	0.479	0.455	0.479
		0.99	5.366	5.735	5.232	5.591	5.206	5.563	5.366	5.735	5.232	5.591	5.167	5.521	5.366	5.735	5.232	5.591	5.129	5.481

0.9	1	0.7	0.175	0.185	0.165	0.175	0.163	0.173	0.175	0.185	0.165	0.175	0.161	0.170	0.175	0.185	0.165	0.175	0.159	0.168
		0.8	0.276	0.293	0.251	0.266	0.247	0.262	0.276	0.293	0.251	0.266	0.241	0.255	0.276	0.293	0.251	0.266	0.236	0.250
		0.9	0.543	0.573	0.437	0.462	0.422	0.446	0.543	0.573	0.437	0.462	0.402	0.424	0.543	0.573	0.437	0.462	0.385	0.406
		0.99	6.136	6.416	1.513	1.589	1.329	1.395	6.136	6.416	1.513	1.589	1.129	1.183	6.136	6.416	1.513	1.589	0.986	1.032
	5	0.7	0.150	0.158	0.149	0.158	0.149	0.158	0.150	0.158	0.149	0.158	0.149	0.158	0.150	0.158	0.149	0.158	0.149	0.157
		0.8	0.229	0.242	0.228	0.241	0.228	0.241	0.229	0.242	0.228	0.241	0.228	0.240	0.229	0.242	0.228	0.241	0.227	0.240
		0.9	0.495	0.524	0.490	0.519	0.489	0.518	0.495	0.524	0.490	0.519	0.488	0.517	0.495	0.524	0.490	0.519	0.486	0.515
		0.99	5.646	5.955	4.911	5.178	4.788	5.049	5.646	5.955	4.911	5.178	4.617	4.868	5.646	5.955	4.911	5.178	4.459	4.701
	10	0.7	0.156	0.164	0.156	0.164	0.155	0.163	0.156	0.164	0.156	0.164	0.155	0.163	0.156	0.164	0.156	0.164	0.155	0.163
		0.8	0.229	0.243	0.229	0.243	0.229	0.243	0.229	0.243	0.229	0.243	0.229	0.243	0.229	0.243	0.229	0.243	0.229	0.243
		0.9	0.457	0.480	0.456	0.479	0.455	0.479	0.457	0.480	0.456	0.479	0.455	0.478	0.457	0.480	0.456	0.479	0.455	0.478
		0.99	5.366	5.735	5.195	5.551	5.162	5.516	5.366	5.735	5.195	5.551	5.113	5.464	5.366	5.735	5.195	5.551	5.066	5.413

**Table 17:** Estimated MSEs of OLS, MRE, MRE, MRT, and RTMME at *n* = 100 and *p* = 7 when there is one outlier.

*k*	Sigma	Rho	*d* = 0.2						*d* = 0.5						*d* = 0.8					
			$\hat{\alpha }$	${\hat{\alpha }}_{M}$	$\hat{\alpha }\left(k\right)$	${\hat{\alpha }}_{M}\left(k\right)$	${\hat{\alpha }}_{\text{MRT}}$	${\hat{\alpha }}_{\text{RTMME}}$	$\hat{\alpha }$	${\hat{\alpha }}_{M}$	$\hat{\alpha }\left(k\right)$	${\hat{\alpha }}_{M}\left(k\right)$	${\hat{\alpha }}_{\text{MRT}}$	${\hat{\alpha }}_{\text{RTMME}}$	$\hat{\alpha }$	${\hat{\alpha }}_{M}$	$\hat{\alpha }\left(k\right)$	${\hat{\alpha }}_{M}\left(k\right)$	${\hat{\alpha }}_{\text{MRT}}$	${\hat{\alpha }}_{\text{RTMME}}$
0.3	1	0.7	0.414	0.187	0.407	0.184	0.406	0.183	0.414	0.187	0.407	0.184	0.404	0.182	0.414	0.187	0.407	0.184	0.402	0.181
		0.8	0.606	0.295	0.591	0.285	0.588	0.284	0.606	0.295	0.591	0.285	0.583	0.281	0.606	0.295	0.591	0.285	0.579	0.278
		0.9	1.155	0.578	1.092	0.534	1.081	0.526	1.155	0.578	1.092	0.534	1.065	0.515	1.155	0.578	1.092	0.534	1.049	0.504
		0.99	12.070	6.477	7.416	3.111	6.944	2.842	12.070	6.477	7.416	3.111	6.345	2.521	12.070	6.477	7.416	3.111	5.843	2.270
	5	0.7	0.211	0.160	0.211	0.160	0.211	0.160	0.211	0.160	0.211	0.160	0.211	0.160	0.211	0.160	0.211	0.160	0.211	0.160
		0.8	0.316	0.246	0.316	0.245	0.315	0.245	0.316	0.246	0.316	0.245	0.315	0.245	0.316	0.246	0.316	0.245	0.315	0.245
		0.9	0.662	0.531	0.661	0.529	0.660	0.529	0.662	0.531	0.661	0.529	0.660	0.529	0.662	0.531	0.661	0.529	0.659	0.528
		0.99	7.190	6.018	6.898	5.731	6.843	5.678	7.190	6.018	6.898	5.731	6.763	5.599	7.190	6.018	6.898	5.731	6.685	5.523
	10	0.7	0.199	0.168	0.199	0.168	0.199	0.168	0.199	0.168	0.199	0.168	0.199	0.168	0.199	0.168	0.199	0.168	0.199	0.168
		0.8	0.295	0.249	0.295	0.249	0.295	0.249	0.295	0.249	0.295	0.249	0.295	0.249	0.295	0.249	0.295	0.249	0.295	0.249
		0.9	0.603	0.499	0.603	0.498	0.602	0.498	0.603	0.499	0.603	0.498	0.602	0.498	0.603	0.499	0.603	0.498	0.602	0.498
		0.99	6.738	5.870	6.666	5.806	6.651	5.794	6.738	5.870	6.666	5.806	6.630	5.775	6.738	5.870	6.666	5.806	6.609	5.757

0.7	1	0.7	0.414	0.187	0.399	0.179	0.396	0.177	0.414	0.187	0.399	0.179	0.392	0.175	0.414	0.187	0.399	0.179	0.388	0.173
		0.8	0.606	0.295	0.572	0.274	0.566	0.270	0.606	0.295	0.572	0.274	0.557	0.265	0.606	0.295	0.572	0.274	0.549	0.260
		0.9	1.155	0.578	1.023	0.487	1.002	0.473	1.155	0.578	1.023	0.487	0.973	0.453	1.155	0.578	1.023	0.487	0.946	0.436
		0.99	12.070	6.477	5.119	1.933	4.612	1.713	12.070	6.477	5.119	1.933	4.004	1.467	12.070	6.477	5.119	1.933	3.528	1.286
	5	0.7	0.211	0.160	0.211	0.160	0.211	0.160	0.211	0.160	0.211	0.160	0.211	0.160	0.211	0.160	0.211	0.160	0.210	0.160
		0.8	0.316	0.246	0.315	0.245	0.315	0.245	0.316	0.246	0.315	0.245	0.315	0.245	0.316	0.246	0.315	0.245	0.314	0.244
		0.9	0.662	0.531	0.658	0.527	0.657	0.526	0.662	0.531	0.658	0.527	0.656	0.525	0.662	0.531	0.658	0.527	0.655	0.524
		0.99	7.190	6.018	6.551	5.393	6.439	5.285	7.190	6.018	6.551	5.393	6.280	5.132	7.190	6.018	6.551	5.393	6.131	4.988
	10	0.7	0.199	0.168	0.199	0.168	0.199	0.168	0.199	0.168	0.199	0.168	0.199	0.168	0.199	0.168	0.199	0.168	0.199	0.168
		0.8	0.295	0.249	0.295	0.249	0.295	0.248	0.295	0.249	0.295	0.249	0.295	0.248	0.295	0.249	0.295	0.249	0.295	0.248
		0.9	0.603	0.499	0.602	0.498	0.602	0.498	0.603	0.499	0.602	0.498	0.602	0.497	0.603	0.499	0.602	0.498	0.601	0.497
		0.99	6.738	5.870	6.572	5.724	6.539	5.696	6.738	5.870	6.572	5.724	6.492	5.654	6.738	5.870	6.572	5.724	6.445	5.613

0.9	1	0.7	0.163	0.173	0.414	0.187	0.395	0.177	0.414	0.187	0.395	0.177	0.387	0.172	0.414	0.187	0.395	0.177	0.382	0.170
		0.8	0.247	0.262	0.606	0.295	0.564	0.268	0.606	0.295	0.564	0.268	0.546	0.258	0.606	0.295	0.564	0.268	0.536	0.252
		0.9	0.422	0.446	1.155	0.578	0.993	0.467	1.155	0.578	0.993	0.467	0.935	0.429	1.155	0.578	0.993	0.467	0.905	0.411
		0.99	1.329	1.395	12.070	6.477	4.422	1.634	12.070	6.477	4.422	1.634	3.354	1.222	12.070	6.477	4.422	1.634	2.915	1.068
	5	0.7	0.149	0.158	0.211	0.160	0.211	0.160	0.211	0.160	0.211	0.160	0.210	0.160	0.211	0.160	0.211	0.160	0.210	0.160
		0.8	0.228	0.241	0.316	0.246	0.315	0.245	0.316	0.246	0.315	0.245	0.314	0.244	0.316	0.246	0.315	0.245	0.314	0.244
		0.9	0.489	0.518	0.662	0.531	0.657	0.526	0.662	0.531	0.657	0.526	0.654	0.523	0.662	0.531	0.657	0.526	0.652	0.522
		0.99	4.788	5.049	7.190	6.018	6.393	5.241	7.190	6.018	6.393	5.241	6.069	4.930	7.190	6.018	6.393	5.241	5.894	4.762
	10	0.7	0.155	0.163	0.199	0.168	0.199	0.168	0.199	0.168	0.199	0.168	0.199	0.168	0.199	0.168	0.199	0.168	0.199	0.168
		0.8	0.229	0.243	0.295	0.249	0.295	0.248	0.295	0.249	0.295	0.248	0.294	0.248	0.295	0.249	0.295	0.248	0.294	0.248
		0.9	0.455	0.479	0.603	0.499	0.602	0.498	0.603	0.499	0.602	0.498	0.601	0.497	0.603	0.499	0.602	0.498	0.601	0.497
		0.99	5.162	5.516	6.738	5.870	6.526	5.684	6.738	5.870	6.526	5.684	6.425	5.596	6.738	5.870	6.526	5.684	6.366	5.544

**Table 18:** Estimated MSEs of OLS, MRE, MRE, MRT, and RTMME at *n* = 100 and *p* = 7 when there are two outliers.

*k*	Sigma	Rho	*d* = 0.2						*d* = 0.5						*d* = 0.8					
			$\hat{\alpha }$	${\hat{\alpha }}_{M}$	$\hat{\alpha }\left(k\right)$	${\hat{\alpha }}_{M}\left(k\right)$	${\hat{\alpha }}_{\text{MRT}}$	${\hat{\alpha }}_{\text{RTMME}}$	$\hat{\alpha }$	${\hat{\alpha }}_{M}$	$\hat{\alpha }\left(k\right)$	${\hat{\alpha }}_{M}\left(k\right)$	${\hat{\alpha }}_{\text{MRT}}$	${\hat{\alpha }}_{\text{RTMME}}$	$\hat{\alpha }$	${\hat{\alpha }}_{M}$	$\hat{\alpha }\left(k\right)$	${\hat{\alpha }}_{M}\left(k\right)$	${\hat{\alpha }}_{\text{MRT}}$	${\hat{\alpha }}_{\text{RTMME}}$
0.3	1	0.7	0.835	0.192	0.827	0.188	0.825	0.188	0.835	0.192	0.827	0.188	0.823	0.187	0.835	0.192	0.827	0.188	0.820	0.186
		0.8	1.220	0.302	1.200	0.292	1.196	0.290	1.220	0.302	1.200	0.292	1.191	0.288	1.220	0.302	1.200	0.292	1.185	0.285
		0.9	2.304	0.589	2.220	0.546	2.204	0.538	2.304	0.589	2.220	0.546	2.181	0.527	2.304	0.589	2.220	0.546	2.158	0.516
		0.99	23.201	6.580	15.780	3.202	14.892	2.929	23.201	6.580	15.780	3.202	13.730	2.604	23.201	6.580	15.780	3.202	12.725	2.348
	5	0.7	0.264	0.163	0.264	0.163	0.264	0.163	0.264	0.163	0.264	0.163	0.264	0.163	0.264	0.163	0.264	0.163	0.264	0.163
		0.8	0.401	0.251	0.400	0.250	0.400	0.250	0.401	0.251	0.400	0.250	0.400	0.250	0.401	0.251	0.400	0.250	0.399	0.250
		0.9	0.830	0.540	0.827	0.538	0.826	0.538	0.830	0.540	0.827	0.538	0.825	0.537	0.830	0.540	0.827	0.538	0.824	0.537
		0.99	8.931	6.102	8.546	5.810	8.474	5.755	8.931	6.102	8.546	5.810	8.369	5.675	8.931	6.102	8.546	5.810	8.266	5.598
	10	0.7	0.311	0.178	0.311	0.178	0.311	0.178	0.311	0.178	0.311	0.178	0.311	0.178	0.311	0.178	0.311	0.178	0.311	0.178
		0.8	0.463	0.265	0.463	0.265	0.463	0.265	0.463	0.265	0.463	0.265	0.463	0.265	0.463	0.265	0.463	0.265	0.463	0.265
		0.9	0.949	0.537	0.948	0.537	0.948	0.537	0.949	0.537	0.948	0.537	0.948	0.537	0.949	0.537	0.948	0.537	0.948	0.537
		0.99	10.099	6.192	10.000	6.126	9.980	6.113	10.099	6.192	10.000	6.126	9.951	6.094	10.099	6.192	10.000	6.126	9.921	6.075

0.7	1	0.7	0.835	0.192	0.816	0.184	0.813	0.182	0.835	0.192	0.816	0.184	0.807	0.180	0.835	0.192	0.816	0.184	0.802	0.179
		0.8	1.220	0.302	1.175	0.281	1.167	0.277	1.220	0.302	1.175	0.281	1.155	0.272	1.220	0.302	1.175	0.281	1.143	0.267
		0.9	2.304	0.589	2.120	0.499	2.088	0.485	2.304	0.589	2.120	0.499	2.042	0.466	2.304	0.589	2.120	0.499	1.998	0.450
		0.99	23.201	6.580	11.231	2.004	10.155	1.779	23.201	6.580	11.231	2.004	8.836	1.526	23.201	6.580	11.231	2.004	7.779	1.339
	5	0.7	0.264	0.163	0.264	0.163	0.264	0.162	0.264	0.163	0.264	0.163	0.263	0.162	0.264	0.163	0.264	0.163	0.263	0.162
		0.8	0.401	0.251	0.399	0.250	0.399	0.250	0.401	0.251	0.399	0.250	0.398	0.249	0.401	0.251	0.399	0.250	0.398	0.249
		0.9	0.830	0.540	0.823	0.536	0.822	0.535	0.830	0.540	0.823	0.536	0.820	0.533	0.830	0.540	0.823	0.536	0.818	0.532
		0.99	8.931	6.102	8.092	5.466	7.947	5.356	8.931	6.102	8.092	5.466	7.741	5.200	8.931	6.102	8.092	5.466	7.548	5.053
	10	0.7	0.311	0.178	0.311	0.178	0.311	0.178	0.311	0.178	0.311	0.178	0.311	0.178	0.311	0.178	0.311	0.178	0.311	0.178
		0.8	0.463	0.265	0.462	0.265	0.462	0.265	0.463	0.265	0.462	0.265	0.462	0.265	0.463	0.265	0.462	0.265	0.462	0.265
		0.9	0.949	0.537	0.948	0.536	0.947	0.536	0.949	0.537	0.948	0.536	0.947	0.536	0.949	0.537	0.948	0.536	0.946	0.536
		0.99	10.099	6.192	9.870	6.041	9.826	6.012	10.099	6.192	9.870	6.041	9.760	5.969	10.099	6.192	9.870	6.041	9.695	5.926

0.9	1	0.7	0.835	0.192	0.811	0.182	0.807	0.180	0.835	0.192	0.811	0.182	0.800	0.178	0.835	0.192	0.811	0.182	0.794	0.176
		0.8	1.220	0.302	1.163	0.276	1.153	0.271	1.220	0.302	1.163	0.276	1.138	0.265	1.220	0.302	1.163	0.276	1.123	0.260
		0.9	2.304	0.589	2.074	0.480	2.035	0.464	2.304	0.589	2.074	0.480	1.980	0.443	2.304	0.589	2.074	0.480	1.929	0.424
		0.99	23.201	6.580	9.746	1.698	8.670	1.496	23.201	6.580	9.746	1.698	7.389	1.273	23.201	6.580	9.746	1.698	6.394	1.113
	5	0.7	0.264	0.163	0.264	0.162	0.263	0.162	0.264	0.163	0.264	0.162	0.263	0.162	0.264	0.163	0.264	0.162	0.263	0.162
		0.8	0.401	0.251	0.399	0.249	0.398	0.249	0.401	0.251	0.399	0.249	0.398	0.249	0.401	0.251	0.399	0.249	0.397	0.249
		0.9	0.830	0.540	0.821	0.534	0.819	0.533	0.830	0.540	0.821	0.534	0.817	0.531	0.830	0.540	0.821	0.534	0.814	0.530
		0.99	8.931	6.102	7.886	5.310	7.712	5.178	8.931	6.102	7.886	5.310	7.469	4.994	8.931	6.102	7.886	5.310	7.244	4.824
	10	0.7	0.311	0.178	0.311	0.178	0.311	0.178	0.311	0.178	0.311	0.178	0.311	0.178	0.311	0.178	0.311	0.178	0.311	0.178
		0.8	0.463	0.265	0.462	0.265	0.462	0.265	0.463	0.265	0.462	0.265	0.462	0.265	0.463	0.265	0.462	0.265	0.462	0.265
		0.9	0.949	0.537	0.947	0.536	0.947	0.536	0.949	0.537	0.947	0.536	0.946	0.536	0.949	0.537	0.947	0.536	0.946	0.535
		0.99	10.099	6.192	9.807	6.000	9.750	5.963	10.099	6.192	9.807	6.000	9.667	5.908	10.099	6.192	9.807	6.000	9.585	5.855

**Table 19:** Estimated MSE values of the OLSE, ME, RE, RME, MRT, and RTMME in Hussein data set.

Coefficients	*α*	${\hat{\alpha }}_{M}$	$\hat{\alpha }\left(k\right)$	${\hat{\alpha }}_{M}\left(k_{\text{HM}}\right)$	${\hat{\alpha }}_{\text{MRT}}\left(k_{\text{HMR}},\;d\right)$	${\hat{\alpha }}_{\text{RTMME}}\left(\left({\tilde{k}}_{\text{HMR}},\;d\right.\right)$
*β*_0_	208.874	173.331	178.6184	155.4684	178.6184	155.4684
*β*_1_	−2.0171	−1.8846	−2.0167	−1.8844	−2.0167	−1.8844
*β*_2_	1.51533	1.6340	1.5153	1.6339	1.5153	1.6339
*β*_3_	−1.3143	−1.3527	−1.3143	−1.3527	−1.3143	−1.3527
MSE	20875.62	9751.86	2269.33	1952.60	2269.33	629.58

*k*=1.9136,  *k*_HM_=1.2980,  *k*_HMR_=0.9813,  *k*_HMR_=0.6656 , and *d*=0.95.

## Data Availability

The data used to support the findings of this study are available in page 7 of [19].
